# Internet‐administered, low‐intensity cognitive behavioral therapy for parents of children treated for cancer: A feasibility trial (ENGAGE)

**DOI:** 10.1002/cam4.5377

**Published:** 2022-11-20

**Authors:** Ella Thiblin, Joanne Woodford, Christina Reuther, Johan Lundgren, Nina Lutvica, Louise von Essen

**Affiliations:** ^1^ Healthcare Sciences and e‐Health Department of Women's and Children's Health Uppsala University Uppsala Sweden

**Keywords:** anxiety, cancer, cognitive behavioral therapy, depression, internet‐based intervention, parents

## Abstract

**Background:**

Parents of children treated for cancer may experience mental health difficulties, such as depression and anxiety. There is a lack of evidence‐based psychological interventions for parents, with psychological support needs unmet. An internet‐administered, guided, low‐intensity cognitive behavioral therapy‐based (LICBT) self‐help intervention may provide a solution.

**Methods:**

The feasibility and acceptability of such an intervention was examined using a single‐arm feasibility trial (ENGAGE). Primary objectives examined: (1) estimates of recruitment and retention rates; (2) feasibility and acceptability of data collection instruments and procedures; and (3) intervention feasibility and acceptability. Clinical outcomes were collected at baseline, post‐treatment (12 weeks), and follow‐up (6 months).

**Results:**

The following progression criteria were met: sample size was exceeded within 5 months, with 11.0% enrolled of total population invited, study dropout rate was 24.0%, intervention dropout was 23.6%, missing data remained at ≤10% per measure, and no substantial negative consequences related to participation were reported. Intervention adherence was slightly lower than progression criteria (47.9%).

**Conclusion:**

Findings suggest an internet‐administered, guided, LICBT self‐help intervention may represent a feasible and acceptable solution for parents of children treated for cancer. With minor study protocol and intervention modifications, progression to a pilot randomized controlled trial (RCT) and subsequent superiority RCT is warranted.

## Abstract 2

A single‐arm feasibility trial showed overall acceptability and feasibility of an internet‐administered, guided, low‐intensity cognitive behavioral therapy intervention for parents of childhood cancer survivors, and progression criteria were met for recruitment, retention, missing data, and harms, indicating methods, study procedures, and the overall the trial and intervention was feasible and acceptable. However, completion of assessments at each timepoint and intervention adherence were under progression criteria, meaning some modifications to the study protocol intervention are required before commencing a pilot randomized controlled trial.

## 1 INTRODUCTION

Advances in cancer treatment have resulted in increased childhood cancer survival rates worldwide.^1^, ^2^ Parents are the primary source of support for children with cancer, with many actively involved in care years after treatment completion.^3^ While treatment completion is an important milestone, it is also a period of vulnerability for parents.^4^, ^5^ Psychological difficulties such as anxiety (19.7% to 43.4%),^6^, ^7^ and depression (14.4% to 43.4%)^6^, ^7^ are reported. Parents also report post‐traumatic stress symptoms^8^, ^9^ and 19.1% of mothers and 7.8% of fathers report at least partial post‐traumatic stress disorder (PTSD) 5 years after treatment.^10^ Further, parents face socioeconomic impacts^11^ and restrictions on daily life activities.^12^ However, parents' psychological needs are unmet^13^ and barriers to seeking support include lack of time, guilt, and putting the child's needs first.^14^, ^15^

Solutions to increase access to psychological interventions are being implemented globally,^16^ for example low‐intensity cognitive behavioral therapy (LICBT).^17^ LICBT is delivered through self‐help interventions (e.g., print or digital format), including internet‐administered CBT (iCBT)^18^ rather than by traditional psychologists. Guided iCBT (supported by a trained professional) is associated with higher effect sizes than unguided interventions^19^ and show equivalent overall effects to traditional face‐to‐face interventions.^20^ An internet‐administered, guided, LICBT based self‐help intervention may also address barriers to seeking support given increased privacy and flexibility.^21^ In previous research, we have shown that an iCBT self‐help intervention decreases symptoms of anxiety, depression, and post‐traumatic stress in parents of children on cancer treatment.^22^, ^23^ Recent research has also demonstrated a video‐conference‐based internet‐administered intervention to be effective for parents of children living with a life‐threatening illness (including cancer).^24^ However, to the best of our knowledge, the only existing internet‐administered intervention for parents of children who have completed treatment, with published results, is an online group‐based, intervention delivered in real time via videoconferencing by psychologists.^25^, ^26^ As such, there is currently no internet‐administered, LICBT based self‐help intervention available for parents of children who have completed cancer treatment.

A program of phase I (development) research, following the Medical Research Council complex interventions framework^27^, ^28^ informed development of the internet‐administered LICBT intervention EJDeR.^10^, ^29^, ^30^, ^31^, ^32^, ^33^, ^34^ Following phase II (feasibility)^27^, ^28^ we conducted the current study, the single‐arm feasibility trial ENGAGE. Primary objectives examined methodological, procedural, and clinical uncertainties^35^ to prepare for the design and conduct of a future pilot RCT and subsequent superiority RCT. Information was gathered on: (1) estimates of recruitment and retention rates; (2) feasibility and acceptability of data collection instruments and procedures; and (3) feasibility and acceptability of the intervention. An embedded mixed‐method process evaluation examined the feasibility of collecting weekly assessments and semi‐structured interviews at baseline and post‐treatment explored: (1) self‐reported psychological concerns, healthcare utilization, and productivity losses; (2) treatment expectations; (3) intervention acceptability; and (4) perceived impact of the intervention on difficulties and mechanisms of change. Findings from semi‐structured interviews at baseline and post‐treatment to inform the embedded process evaluation will be reported elsewhere.

## 2 MATERIALS AND METHODS

The study protocol is published^36^ and registered, with results following the Consolidated Standards of Reporting Trials (CONSORT) 2010 statement extension for randomized pilot and feasibility trials.^37^

### 2.1 Study design

A single‐arm feasibility trial of a guided, internet‐administered LICBT‐based intervention (EJDeR), with data collected at baseline, post‐treatment (12 weeks), and follow‐up (6 months) with an embedded mixed‐methods process evaluation. EJDeR is delivered via the U‐CARE‐portal (Portal), a web‐based platform, designed to deliver internet‐administered interventions and support the execution of study procedures.

### 2.2 Participants

Eligible participants were: (1) parent of a child diagnosed with childhood cancer (0–18 years) who completed treatment 3 months to 5 years previously (timespan informed by our previous longitudinal research that has identified this as a time period of vulnerability for parents)^9^, ^10^, ^29^, ^30^; (2) resident in Sweden; (3) able to read and understand Swedish; (4) able to access e‐mail, internet, and Bank‐ID (a Swedish citizen authentication system); and (5) self‐reporting a need for psychological support related to the child's cancer. Exclusion criteria were: (1) a self‐reported or clinician assessed (with the Mini‐International Neuropsychiatric Interview, M.I.N.I., version 7.0.0)^38^ severe and enduring mental health difficulty (e.g., PTSD) and/or misuse of alcohol, street drugs, or prescription medication; (2) acute suicidality; and (3) ongoing psychological treatment respectively.

### 2.3 Recruitment

#### 2.3.1 Postal study invitations

Personal identification numbers of children who had completed treatment 3 months to 5 years previously were provided by the Swedish Childhood Cancer Registry (CCR), and linked to parents' names and addresses via NAVET, a population registry from the Swedish Tax Agency. The first recruitment block was pre‐selected with parents of children who had ended treatment near to 5 years previously. The following four blocks were randomly selected by a member of the Portal team, independent to the research team, using a computer‐generated simple randomization procedure. Postal study invitation packs, sent to parents' home addresses, included a: (1) study invitation letter; (2) study information sheet and link to a secure website on the Portal (information in text and video format); (3) paper reply‐slip to register interest in participation; (4) paper‐based opt‐out form and reasons for non‐participation questionnaire; and (5) freepost envelope. Parents could register interest in participation and request more study information via the Portal, post, telephone or e‐mail. ENGAGE included an embedded recruitment RCT, investigating the effect of personalized versus non‐personalized study invitations on recruitment and retention^39^ with results reported separately.^40^

#### 2.3.2 Online advertisements

Advertisements were placed on social media sites, websites, and newsletters of 12 cancer organizations and interest groups.

#### 2.3.3 Opt‐out and reminders

Parents invited via the post could opt‐out of ENGAGE via the Portal, post, telephone, or e‐mail. Up to five reminder telephone contact attempts were made if parents did not respond within 4 weeks of invitation. Telephone numbers were identified using internet search engines. Contact attempts were documented in paper‐based case report forms (CRFs). If a telephone number was not identified, a postal study invitation reminder letter was sent.

#### 2.3.4 Reasons for non‐participation

Parents opting out of ENGAGE were asked to complete a reason for non‐participation questionnaire including a closed, multiple choice question and an open question for other reason(s).^41^ Reasons for non‐participation were collected to enable the identification of potential modifiable barriers to participation (e.g., treatment preferences, interest in internet‐administered self‐help, burden of trial procedures).

### 2.4 Consent, eligibility, and baseline

Parents provided consent via the Portal. Parents who registered interest in participation but did not provide consent, or opt out, within 2 weeks, were contacted to confirm interest in participation (maximum five reminders via telephone, SMS or e‐mail).

Parents providing consent were contacted to organize a telephone eligibility interview with a licensed psychologist. Interviews included: (1) questions concerning eligibility criteria; with those eligible completing specific modules of the M.I.N.I., and; (2) questions concerning parent and child sociodemographic and clinical characteristics (Table 1).

**TABLE 1:** Overview of measures taken at respective assessment time‐point

Variable	Measure	Time‐point					Mode of administration
		Eligibility interview	Baseline	Post‐treatment	Weekly process evaluation	Follow‐up	
Child age, legal gender, cancer diagnosis, date of first diagnosis, date of end of treatment (where available), type of treatment	Childhood Cancer Registry						Swedish Childhood Cancer Registry
Eligibility (inclusion and exclusion) criteria; parent sociodemographic and clinical characteristics (age, gender, relationship status, highest level of education, employment status, number and ages of children, current housing situation, region of birth, previous psychological treatment, physical health problem, previous traumatic/difficult life events and internet usage); child sociodemographic and clinical characteristics (age, gender, cancer diagnosis, time since end of treatment, type of treatment, cancer recurrence)	Structured questions	✓					Telephone
Psychiatric (mood and anxiety) disorders, drug and alcohol misuse, suicidality	M.I.N.I.	✓		✓		✓	Telephone
PTSS	PCL‐5		✓	✓	✓	✓	Portal/Telephone Weekly process evaluation: Portal only
PTSS	PCL‐C		✓	✓	✓	✓	Portal/Telephone Weekly process evaluation: Portal only
Depression	PHQ‐9		✓	✓	✓	✓	Portal/Telephone Weekly process evaluation: Portal only
Anxiety	GAD‐7		✓	✓		✓	Portal/Telephone
Fear of recurrence	FRHC		✓	✓		✓	Portal/Telephone
Fear of serious health condition	FRHC		✓	✓		✓	Portal/Telephone
Psychological inflexibility and experiential avoidance	AAQ‐6		✓	✓	✓	✓	Portal/Telephone Weekly process evaluation: Portal only
Depressed inactivity	BADS		✓	✓	✓	✓	Portal/Telephone Weekly process evaluation: Portal only
Fatigue	FSS		✓	✓		✓	Portal/Telephone
Quality of life	EQ‐5D		✓	✓		✓	Portal/Telephone
Self‐compassion	SCS‐SF		✓	✓		✓	Portal/Telephone
Health economics	TIC‐P		✓	✓			Portal/Telephone

Abbreviations: AAQ‐6, Acceptance and Action Questionnaire; BADS, Behavioral Activation for Depression Scale; EQ‐5D, EuroQol 5‐dimension questionnaire; FRHC, Fear of recurrence and serious health condition (structured questions); FSS, Fatigue Severity Scale; GAD‐7, Generalized Anxiety Disorder 7‐item scale; M.I.N.I., Mini‐International Neuropsychiatric Interview version 7.0.0; PCL‐5, Post‐traumatic Stress Disorder Checklist for DSM‐5; PCL‐C, adapted version of Post‐traumatic Stress Disorder Checklist‐Civilian version; PHQ‐9, Patient Health Questionnaire; PTSS, Post‐traumatic stress symptoms; SCS‐SF, Self‐Compassion Scale‐Short Form; TIC‐P, Treatment Inventory of Costs in Patients with psychiatric disorders.

Eligible participants were enrolled and invited to an optional semi‐structured telephone interview with a licensed psychologist to explore concerns, needs, healthcare utilization, and productivity loss, alongside expectations on the trial and intervention. Participants gained access to the Portal assessment at baseline (Table 1) and were required to complete the assessment within 28 days. Participants who had not completed within 14 days were reminded up to five times (telephone, SMS, or e‐mail). Upon completion of the Portal assessment at baseline, participants gained access to EJDeR and were allocated to an e‐therapist.

### 2.5 Intervention

The EJDeR protocol is published following the Template for Intervention Description and Replication (TIDieR) checklist.^34^, ^42^ The first version of the intervention used a multi‐strand approach utilizing several CBT techniques, including third‐wave CBT (e.g., mindfulness and compassion focused therapy), delivered over 10 modules.^32^, ^36^ Following public and professional involvement^34^ the number of CBT techniques were minimized to reduce complexity and length^34^ and a LICBT approach was adopted.

EJDeR is a guided internet‐administered LICBT intervention delivered over 12 weeks on the Portal and includes text, illustrations, film, audio files, in‐module exercises, and homework exercises. EJDeR includes two LICBT techniques: behavioral activation (BA) for depression, and worry management (WM) for generalized anxiety disorder (GAD).^34^ It consists of four modules: (1) introduction and psychoeducation; (2) BA; (3) WM, and; (4) relapse prevention (Figure S1). After completing the first module and an initial assessment session with an e‐therapist, participants work with BA or WM, dependent on their main difficulty. After completion of BA or WM, parents may use the remaining LICBT technique. All participants gain access to the relapse prevention module.

E‐therapist guidance is provided via an initial assessment session (video‐conferencing or telephone, ≈45 min); weekly support via written messages via the Portal (≈20–30 min/week), and a mid‐intervention booster session (video‐conferencing or telephone, ≈30–45 min) following structured protocols.^43^, ^44^, ^45^ E‐therapists also provided at‐need written messages to participants if requested. A 2‐day training program with two experts in LICBT, and weekly group clinical supervision via video‐conferencing with a Swedish licensed psychologist with expertise in iCBT were provided.

### 2.6 Outcomes

Feasibility outcomes are informed by the CONSORT 2010 statement extension for randomized pilot and feasibility trials,^35^, ^37^ and relate to methodological uncertainties (e.g. estimates of recruitment and retention rates, reasons for non‐participation and study drop‐out), procedural uncertainties (e.g. feasibility and acceptability of data collection instruments and procedures, including percentages completing assessments and numbers of missing items), and clinical uncertainties (e.g. intervention feasibility and acceptability, including participants' adherence to the intervention and impressions and experiences of working with the intervention). All feasibility outcomes are shown in Table 2 alongside progression criteria.^46^ Some feasibility outcomes^36^ were revised to improve clarity and reflect protocol modifications (Table S1). Intervention acceptability is further explored in the embedded process evaluation (reported elsewhere). Progression criteria were informed by the researchers' previous experience, our previous longitudinal research with the population^6^ and relevant literature on recruitment,^47^, ^48^ attrition,^49^ adherence,^50^, ^51^ and missing data.^52^

**TABLE 2:** Overview of feasibility outcomes, methods of evaluation, and progression criteria

Outcome	Evaluation	Progression criteria to controlled trial^a^
Recruitment and eligibility	Number identified via postal study invitations (Swedish Childhood Cancer Registry and the Swedish Tax Agency [NAVET]) and/or via Online advertisements via cancer organizations and interest groups	No criteria set
	Percentage consented to participate, assessed for eligibility, fulfilling eligibility criteria, and enrolled (of total number invited)	≥9% enrolled of total participant population invited (e.g., included of total participant population invited)
	Reasons for ineligibility	No criteria set
	Ambiguities regarding eligibility criteria including diagnostic uncertainties in M.I.N.I.	No criteria set
	Reasons for non‐participation	No criteria set
Data collection	Percentage completing assessments M.I.N.I. (eligibility interview, post‐treatment, and follow‐up) Semi‐structured interview (baseline and post‐treatment) Portal assessment (baseline, post‐treatment, and follow‐up) Weekly Portal assessment	≥70% answering all questions at all assessments
	Numbers of missing items M.I.N.I. (eligibility interview, post‐treatment, and follow‐up) Portal assessment (baseline, post‐treatment, and follow‐up) Weekly Portal assessment	≤10% per measure
Attrition	Rate of study dropout Rate of intervention dropout	≤30% ≤30%
Resources needed to complete the study and the intervention	Length of time required for: Participants to work through the intervention Participants to complete the initial assessment session and mid‐intervention booster session with e‐therapist Participants to complete the eligibility interview, M.I.N.I., semi‐structured interview, Portal assessment at each time‐point E‐therapists to deliver the intervention	No criteria set
	Number of: Internal and external study personnel Reminder contacts needed during recruitment Reminder contacts needed to complete Portal assessment at each time‐point Contacts needed to arrange eligibility interview, M.I.N.I. and semi‐structured interview over the telephone at each time‐point	No criteria set
Participants' adherence to intervention	Number of: Participants adhering to the minimum treatment dose (MTD) Opened modules Completed LICBT modules started with Completed initial assessment sessions Completed mid‐intervention booster sessions Completed homework sheets	≥50% adhering to MTD, i.e., attending the initial assessment session, completing the introduction and psychoeducation module and one LICBT treatment module (i.e. behavioral activation or worry management) and attending the mid‐intervention booster session.
Participants' use of the intervention	Number of: Participant logins Participant written messages E‐therapist written messages	No criteria set
E‐therapists' adherence to intervention	Content of initial assessment session, mid‐intervention booster session, and written messages via the Portal	No criteria set
Participants' acceptability of the intervention and data collection	Reasons for low adherence and dropout from study and intervention Number of risk assessments Impressions and experiences of working with the intervention (including positive and negative consequences) and of completing assessments and interviews^b^	No criteria set No criteria set <1 participant reporting substantial negative consequences related to participation in the study and/or intervention

Abbreviations: LICBT, low intensity cognitive behavioral therapy; M.I.N.I., Mini‐International Neuropsychiatric Interview version 7.0.0.

^a^ If one or more criteria are not met revisions should be considered before proceeding to a controlled trial.

^b^ Outcome is to be reported in separate publications.

The post‐treatment time‐point was set at 12 weeks, immediately after the EJDeR intervention had finished. A 6‐month follow‐up time‐point was selected to examine the feasibility of longer‐term data collection.

Sociodemographic data on parents and children, specific modules of the M.I.N.I. assessing current and past psychiatric disorders and suicidality, and psychological and health economic measures are reported in Table 1, alongside data collection time‐point and mode of administration. A random 10% sample of M.I.N.I.s were coded by a member of the research team, with inter‐rater reliability calculated as satisfactory (α = 0.92).^53^

Semi‐structured interviews were conducted at baseline and post‐treatment with licensed psychologists (data reported elsewhere).

### 2.7 Sample size

Following recommendations for feasibility trial sample sizes the target sample size was 50.^54^

### 2.8 Double data entry

Paper‐based CRFs were used for data collected outside the Portal, with data independently entered onto a Microsoft® Access database by two research assistants, exported into Microsoft® Excel spreadsheets, with accuracy checked using Microsoft® Spreadsheet.

### 2.9 Reminders

A prompt (SMS and/or e‐mail) was sent when it was time to complete Portal assessments with automatic reminders (SMS and/or e‐mail) sent if not completed within 1 week. Participants who did not complete Portal assessments within 2 weeks, were offered to complete over the telephone, with up to six reminder attempts made via telephone, SMS, or email. Informed by evidence suggesting study newsletters can improve retention^55^ a newsletter was sent via the Portal 6 weeks before post‐treatment and follow‐up.

### 2.10 Participant adherence

The minimum treatment dose (MTD) (i.e., full intervention adherence) was defined as: (1) attendance of the initial assessment session; (2) completion of the introduction and psychoeducation module; (3) completion of one LICBT module (BA or WM); and (4) attendance of the mid‐intervention booster session.

### 2.11 E‐therapist adherence

A 15% random sample of initial assessment and mid‐intervention booster sessions and written messages via the Portal from e‐therapists were marked for adherence, with each item within the structured support protocols marked as absent/present.

### 2.12 Statistical methods

Feasibility outcomes relating to recruitment and eligibility, data collection, attrition, resources needed to complete the study and the intervention, participants' adherence to the intervention, participants' use of the intervention, e‐therapists' adherence to the intervention, and participants' sociodemographic characteristics are reported using descriptive statistics. Numbers and percentages (and 95% CIs where appropriate) are reported for categorical variables, means and SDs for continuous variables. Numbers and percentages of participants meeting criteria for each M.I.N.I. diagnosis is reported at each time‐point. Means and SDs for continuous variables and numbers and percentages for categorical variables are reported for all outcomes at each time‐point. Mean change scores (with 95% CIs) are reported for Portal assessments of psychological outcomes at each time‐point, to describe the study sample.

### 2.13 Risk and safety procedures

Participants scoring >0 on PHQ‐9 (depression) question 9 (suicidal ideation), or a total score >20 (severe depression) were risk assessed by a licensed psychologist within one working day. If needed, participants were directed to appropriate support and excluded.

### 2.14 Public involvement

A Parent Research Partner (PRP) group was established consisting of four parents with lived experience of being a parent of a child treated for cancer (two fathers and two mothers, aged between 45 and 54 years of age). The PRP group was involved in optimizing the acceptability of EJDeR e.g., relevancy, ease of understanding, content, language, and structure.^34^ The group was also consulted on the development of participant invitation letters.^39^, ^40^

## 3 RESULTS

Data supporting feasibility objectives pertaining to recruitment and eligibility, data collection, attrition, and resources needed to complete the study and intervention are available in Zenodo.^56^

### 3.1 Recruitment and eligibility

**FIGURE 1:**
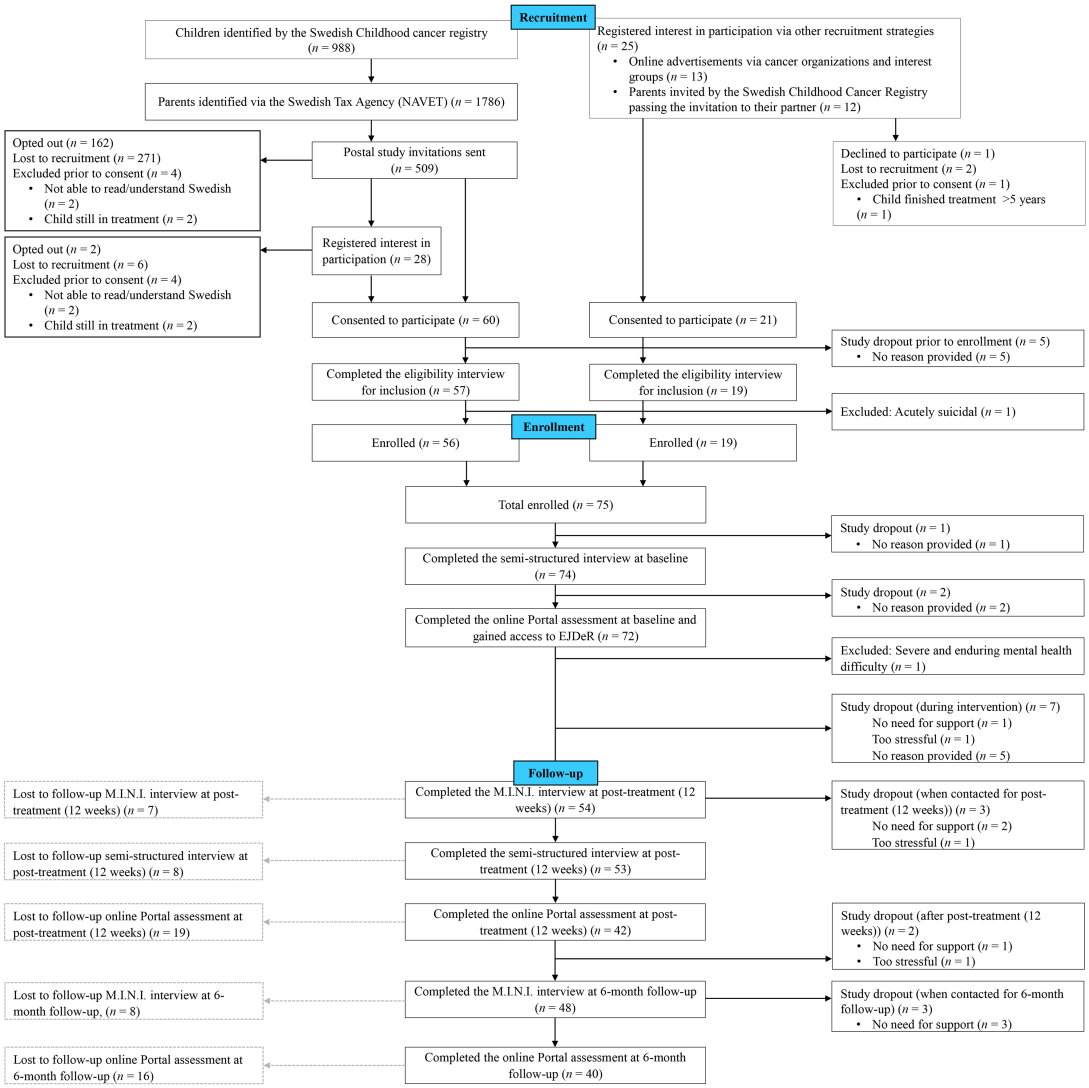
Study flow of participants in the ENGAGE feasibility trial. Solid black lines denote participant flow through the study, including study drop outs i.e., those who discontinued the study. Dashed gray lines represent participants that were lost to follow‐up during assessments at post‐treatment (12 weeks) and follow‐up (6 months) respectively, but had not dropped out of the study.

Participant flow is summarized in an adapted CONSORT diagram (Figure 1). Recruitment took place over 5 months (03‐07‐2020 and 30‐11‐2020). Of 509 study invitations sent via CCR and NAVET, 60 consented (11.8%, 95%CI, [9.1–14.9]); 57 were assessed for eligibility (11.2%, 95%CI, [8.6–14.3]); and 56 fulfilled eligibility criteria and were enrolled (11.0%, 95%CI, [8.4–14.1]) exceeding progression criteria of ≥9% enrolled of total potential participant population invited. An additional 21 consented from other recruitment strategies (online advertisements and parents invited by the CCR passing the invitation to their partner), 19 were assessed for and fulfilled eligibility, and enrolled. Nine parents were excluded prior to consent and one was excluded during the eligibility interview (acute suicidality). In total, 75 participants were enrolled, exceeding sample size expectations (Figure 1).

Ambiguities regarding eligibility arose in six cases. In three cases, parents met criteria for PTSD according to the M.I.N.I. but were included as symptoms were mild. In one case a parent met criteria for Alcohol Use Disorder, and was included due to being in early remission. One was attending a psychological support group; study inclusion was delayed until the group ended. One reported their child had recently relapsed, however, as treatment had not started, the parent was included.

Out of 509 parents identified via the CCR and NAVET, 164 (32.2%) opted out, and 137 provided a response to the multiple‐choice question regarding reasons for non‐participation. Not experiencing any need for psychological support (93/137, 67.9%) was most commonly reported (Table S2). Full results concerning opt‐out rates and reasons for non‐participation have been reported separately.^41^

### 3.2 Sociodemographic and clinical characteristics

Baseline sociodemographic and clinical characteristics for participants (*N* = 75) are summarized in Table 3.

**TABLE 3:** Baseline sociodemographic and clinical self‐report characteristics for participants (*N* = 75)

Sociodemographic and clinical characteristics	*n* (%)
Age (years)	
Mean (SD) range	42.8 (7.1) 26–62
Gender	
Female	48 (64.0)
Male	27 (36.0)
Relationship status	
Partner	63 (84.0)
Single	12 (16.0)
If partner, cohabiting	
Yes	62 (98.4)
No	1 (1.6)
Highest level of education	
Lower secondary	1 (1.3)
Upper secondary	15 (20.0)
Post‐secondary non‐tertiary	3 (4.0)
Tertiary	54 (72.0)
PhD	2 (2.7)
Employment status	
Employed	66 (88.0)
Unemployed	9 (12.0)
Number of children	
Median (range)	2.0 (1–5)
Age of children	
Mean (SD) range	12.0 (7.1) 0.5–37
Housing situation	
Rental	8 (10.7)
Apartment ownership	17 (22.7)
House ownership	47 (62.7)
Other	3 (4.0)
Region of birth	
Nordic countries	63 (84.0)
Asia	6 (8.0)
Europe (excluding. Nordic countries)	5 (6.7)
Africa	1 (1.3)
Previous psychological treatment	
Yes	40 (53.3)
No	35 (46.7)
Physical health problems	
Yes	24 (32.0)
No	51 (68.0)
Type of physical health problem^a^	
Diseases of the musculoskeletal system and connective tissue	9 (12.0)
Endocrine, nutritional and metabolic diseases	5 (6.7)
Diseases of the genitourinary system	3 (4.0)
Diseases of the circulatory system	2 (2.7)
Diseases of the digestive system	2 (2.7)
Diseases of the nervous system	2 (2.7)
Diseases of the respiratory system	2 (2.7)
Diseases of the skin and subcutaneous tissue	1 (1.3)
Neoplasm	1 (1.3)
Other cannot classify	3 (4.0)
Previous traumatic/difficult life event	
Yes	60 (80.0)
No	15 (20.0)
Type pf previous traumatic/difficult life event^a^	
Child's cancer disease	34 (45.3)
Death in family and miscarriage	21 (28.0)
Severe disease/illness own/family/friends	18 (24.0)
Divorce or separation	12 (16.0)
Exposure to violence or sexual abuse	6 (8.0)
Suicide/suicide attempt among family/friends	4 (5.3)
War/terrorist attacks	3 (4.0)
Other traumatic experiences	13 (17.3)

*Note*: Data are number (%) unless stated otherwise. Percentages may not always total 100 due to rounding. Nordic countries represented in the study sample include Denmark, Finland, Norway, and Sweden.

^a^ Multiple responses possible.

Participants' internet usage is reported in Table S10. According to participant self‐report data, children treated for cancer were predominantly male (*n* = 38, 54.3%), had been diagnosed with Leukemia (*n* = 32, 45.7%) and treated with chemotherapy (*n* = 55, 78.6%). The children's mean age at the time of the eligibility interview was 10.6 years (SD 5.2, range, 2–24). Baseline sociodemographic and clinical characteristics for children are provided in Table S10.

### 3.3 Data collection

Data collection (baseline, post‐treatment, and follow‐up) took place between 24‐07‐2020 and 04‐10‐2021. Percentage completing assessments at each time‐point are reported in Table 4, and progression criteria of 70% of participants answering all questions at all assessments was not met. Completion rates of weekly Portal assessments decreased from 65.7% (week one) to 38.9% (week 11) (Table S3).

**TABLE 4:** Number and percentages of participants completing assessments of the total study sample (*N* = 75) and the study sample at baseline, post‐treatment, and follow‐up

Assessment	Completed assessment							
	Total study sample^a^				Study sample at time‐point^b^			
	*N*	*n*	%	95% CI	*N*	*n*	%	95% CI
Baseline								
Semi‐structured interview	75	74	98.7	92.8, 100.0	75	74	98.7	92.8, 100.0
Portal assessment	75	72	96.0	88.8, 99.2	74	72	97.3	90.6, 99.7
Post‐treatment								
M.I.N.I	75	54	72.0	60.4, 81.8	64	54	84.4	73.1, 92.2
Semi‐structured interview	75	53	71.0	59.0, 80.6	64	53	82.8	71.3, 91.1
Portal assessment	75	42	56.0	44.1, 67.5	64	42	65.6	52.7, 77.1
Follow‐up								
M.I.N.I	75	48	64.0	52.1, 74.8	59	48	81.4	69.1, 90.3
Portal assessment	75	40	53.3	41.5, 65.0	59	40	67.8	54.4, 79.4

^a^ Total sample defined as all participants enrolled into the ENGAGE feasibility trial.

^b^ Study sample at time‐point defined as the total number of participants remaining in the ENGAGE feasibility trial (i.e., had not dropped out of, or been excluded from, the study at each time‐point).

Missing data ranged from 0.01%–4.1% items missing per measure, bettering progression criteria (≤10%). Missing data from the M.I.N.I. is reported in Table S4 and missing data for measures included in Portal assessments are reported in Table S5. Missing items for measures included in weekly Portal assessments are provided in Table S6.

### 3.4 Attrition

In total, 18/75 (24.0% [95%CI, 14.9–35.3]) of participants enrolled into the study dropped out of the study, bettering progression criteria (≤30%). In total, 17/72 (23.6% [95%CI, 14.4–35.1]) of participants gaining access to EJDeR, dropped out of EJDeR, bettering progression criteria (≤30%).

### 3.5 Resources needed to complete the study and the intervention

Length of time for participants to work through EJDeR and complete assessments at each time‐point are provided in Table S7. The number of reminder contacts needed during recruitment and for participants to complete Portal assessments are reported in Table S8. The number of contacts needed to arrange interviews at each time‐point are reported in Table S9.

Seventy‐two participants gained access to EJDeR and 71 were allocated to an e‐therapist (one dropped out before allocation). Psychology program students (*n* = 10) supported 27 participants (mean = 2.7, range, 1–7) and spent a mean of 76.9 h (SD 29.7, range, 22.3–109.8) delivering EJDeR, attending training, supervision, and administration, equating to a mean of 2.9 h per participant each week (SD 1.3, range, 0.9–4.6). Due to students not having adequate time to support participant caseloads, the majority were supported by a CBT‐therapist internal to the research team (*n* = 32), a licensed psychologist in the research team (*n* = 5), and a licensed psychologist external to the research team (*n* = 7). The clinical supervisor worked for 155 h, including training, supervision, and administration.

Difficulties recruiting research personnel was identified as a challenge.^57^ The research team included the principal investigator, a researcher, a PhD student/e‐therapist, a research assistant, and an e‐therapist/research assistant. External study personnel included licensed psychologists (*n* = 7) and e‐therapists (*n* = 10). Paper‐based CRFs for study data were considered time and resource intensive, as was coordinating external study personnel.

### 3.6 Participants' adherence to intervention

Seventy‐two participants gained access to EJDeR. One was excluded shortly after access (severe and enduring mental health difficulty) and 34/71 (47.9%) adhered to the MTD, nearly meeting progression criteria of 50%. The mean number of modules opened was 2.3 (SD 0.9, range, 1–4), parents completed a mean of 1.7 modules (SD 1.3, range, 0–4), and a mean of 2.7 homework sheets (SD 2.8, range, 0–11). Initial assessment sessions were attended by 61/71 (85.9%) and mid‐intervention booster sessions were attended by 44/71 (62.0%).

Visual inspection of data indicated differences in adherence rates by first LICBT module started and by gender. A post hoc descriptive analysis was performed. In total, 54/71 (76.1%) started a LICBT module, with 26 starting with BA and 28 with WM. In total, 20/26 (76.9%) starting with BA, and 14/28 (50.0%) starting with WM adhered to the MTD.

Of the 71 participants, 25 were fathers, and 46 were mothers. For fathers: 8/25 (32.0%) started with BA and 7/8 (87.5%) adhered to the MTD; 12/25 (48.0%) started with WM, and 5/12 (41.7%) adhered to the MTD. For mothers, 18/46 (39.1%) started working with BA and 13/18 (72.2%) adhered to the MTD; 16/46 (34.8%) started with WM and 9/16 (56.3%) adhered to the MTD.

### 3.7 Participants' use of the intervention

A mean of 20 participant logins were made (SD 14.9, range, 1–72). A mean of 8.5 participant written messages were sent to e‐therapists (SD 7.6, range, 0–33), and a mean of 28.8 e‐therapist written messages (SD 16.3, range, 0–74) were sent to participants.

### 3.8 E‐therapists' adherence to intervention

Adherence rates were 90.5% for initial assessment sessions, 85.2% for mid‐intervention booster sessions, and 87.5% for written communication between participants and e‐therapists.

### 3.9 Participants' acceptability of the intervention and data collection

Reasons for study dropout are reported in Figure 1. Nineteen risk assessments were conducted and two resulted in study exclusion. No participant reported substantial negative consequences related to study and/or intervention. A structured question asking participants whether the intervention was helpful was omitted by researcher error and it was therefore not possible to assess whether ≥70% of participants using the intervention reported it as helpful (Table 2).

### 3.10 Psychological and health economics outcomes

M.I.N.I. data at baseline, post‐treatment, and follow‐up are provided in Table S12. The mean and SD of outcomes at baseline, post‐treatment, and follow‐up, with 95% CIs, are reported in Table 5, alongside observed changes from baseline to post‐treatment and from baseline to follow‐up (with 95% CI). From baseline to follow‐up depressive symptoms decreased by an average of 3.1 PHQ‐9 points. From baseline to follow‐up anxiety symptoms decreased by an average of 2.9 GAD‐7 points. Descriptive data from the Treatment Inventory of Costs in Patients with psychiatric disorders (TIC‐P) are reported in Table S14. However, due to a large amount of missing data on the TIC‐P it is difficult to interpret this data in a meaningful way.

**TABLE 5:** Treatment outcomes at baseline, post‐treatment, and follow‐up with change scores

Outcome measures	Baseline				Post‐treatment				Follow‐up				Change from baseline to Post‐treatment			Change from baseline to follow‐up		
	*n*	*M*	SD	95% CI^a^	*n*	*M*	SD	95% CI^a^	*n*	*M*	SD	95% CI^a^	*n*	*M*	SD	*n*	*M*	SD
PCL‐5	72	16.0	12.2	10.5, 14.6	42	9.0	8.7	7.2, 11.1	40	5.2	4.8	3.9, 6.2	42	−5.4	6.3	40	−8.2	8.8
PCL‐C	72	31.5	10.9	9.4, 13.0	42	25.1	8.4	6.9, 10.7	40	22.0	4.7	3.9, 6.0	42	−4.9	6.8	40	−7.3	7.9
PHQ‐9	72	6.6	5.0	4.3, 6.0	42	3.8	2.9	2.4, 3.7	40	2.8	2.1	1.7, 2.7	42	−2.0	3.1	40	−3.1	4.7
GAD‐7	72	6.1	4.7	4.0, 5.6	42	3.4	2.9	2.4, 3.7	40	2.6	2.8	2.3, 5.6	42	−2.5	3.9	40	−2.9	4.1
FRHC	72	6.5	1.9	1.6, 2.3	42	5.5	1.5	1.2, 1.9	40	5.0	1.7	1.4, 2.2	42	−0.8	1.5	40	−1.1	1.5
AAQ‐6	72	16.2	8.1	7.0, 9.7	41	12.8	6.8	5.6, 8.7	40	12.4	6.0	4.9, 7.7	41	−2.0	4.4	40	−2.4	5.1
BADS	72	96.1	25.9	22.3, 31.0	42	113.1	16.3	13.4, 20.8	40	118.8	15.2	12.5, 19.5	42	11.5	17.3	40	16.6	22.5
FSS	72	34.2	13.7	11.8, 16.4	42	28.0	10.9	9.0, 13.9	40	26.2	12.3	10.1, 15.8	42	−3.8	7.9	40	−7.0	13.6
EQ‐5D	72	8.1	2.3	2.0, 2.8	42	7.3	1.8	1.5, 2.3	40	6.6	1.5	1.2, 1.9	42	−0.5	1.6	40	−1.0	1.6
EQ‐5D‐VAS	72	66.0	17.2	14.8,20.6	42	74.1	11.2	9.2,14,3	40	77.4	11.7	9.6,15.0	42	6.5	11.6	40	10.4	12.3
SCS‐SF	72	37.8	5.1	4.4, 6.1	42	37.6	7.1	5.8, 9.1	40	37.5	6.3	5.2, 8.1	42	−0.1	6.8	40	−0.6	6.2

Abbreviations: AAQ‐6, Acceptance and Action Questionnaire; BADS, Behavioral Activation for Depression Scale; EQ‐5D, EuroQol 5‐dimension questionnaire; EQ‐5D VAS, EuroQol 5‐dimension questionnaire visual analogue scale; FSS, Fatigue Severity Scale; FRHC, Fear of recurrence and serious health condition (structured questions); GAD‐7, Generalized Anxiety Disorder 7‐item scale; PCL‐5, Post‐traumatic Stress Disorder Checklist for DSM‐5; PCL‐C, adapted version of Post‐traumatic Stress Disorder Checklist‐Civilian version; PHQ‐9, Patient Health Questionnaire; PTSS, Post‐traumatic stress symptoms; SCS‐SF, Self‐Compassion Scale‐Short Form.

^a^ 95% CIs around SD.

## 4 DISCUSSION

The ENGAGE feasibility trial demonstrated it is possible to recruit and retain parents of children treated for cancer into a single‐arm feasibility trial of an internet administered, guided, LICBT based, self‐help intervention. In summary: (1) 12.0% of invited parents consented and 11.0% of invited parents were enrolled, exceeding progression criteria of ≥9%; (2) 24.0% dropped out of the study, and 23.6% dropped out of the intervention, bettering progression criteria of ≤30%; (3) missing items per questionnaire ranged from 0.01% to 3.9%, remaining under ≤10% for all measures, bettering progression criteria; (4) percentage of participants completing assessments ranged from 65.6% to 98.7%, bettering progression criteria of ≥70% for M.I.N.I interviews at all time‐points and Portal assessments at baseline, and marginally under progression criteria of ≥70% for Portal assessments at post‐treatment and follow‐up; (5) intervention adherence was 47.9%, marginally under progression criteria of ≥50%; and (6) no participant reported a substantial negative consequence related to the study and/or intervention, meeting progression criteria. This study was not designed to detect differences in parental depression or anxiety at follow‐up, however reductions in depressive and anxiety symptoms were observed via visual inspection.

### 4.1 Strengths and limitations

To the best of our knowledge, ENGAGE is first trial worldwide designed to test the feasibility of an internet administered, guided, LICBT based, self‐help intervention for parents of children treated for cancer. Robust methods examined a range of feasibility objectives, alongside a priori specified progression criteria. The intervention protocol is published in accordance with TIDieR guidelines^34^ and reporting of methods and results are transparent and complete in accordance with calls for better reporting of feasibility studies.^58^ A novel recruitment strategy was adopted with participants identified via the CCR, meaning invited participants are a nationally representative sample of parents of children treated for cancer. We also successfully adopted an opt‐out recruitment strategy and explored reasons for non‐participation,^41^ which will inform recruitment strategies used in the future pilot RCT. Use of retention strategies, including telephone reminders^59^ and use of a study newsletters^55^ may also have minimized study drop out. Finally, involvement of the PRP group resulted in valuable feedback on intervention content and informed intervention changes, as well as improving study procedures, in line with previous research on the benefits of public involvement in research.^60^, ^61^

The study also has limitations. E‐therapists adherence to the intervention was examined by only one licensed clinical psychologist with adherence marked as absent/present. Future studies should develop an intervention adherence checking tool, examining both adherence and quality.^62^ Participants' adherence to the intervention e.g., the MTD, was defined a priori by the research team and determined by engagement with and use of EJDeR (e.g., module completion). This definition fails to consider activities participants may have engaged in outside of the Portal.^63^ Progression criteria were informed by previous experience and relevant literature. While partly informed by our previous longitudinal research with the population^6^ other literature used to inform the progression criteria include a range of psychological interventions with unique methodological, procedural, and clinical uncertainties. Indeed, lack of clarity on how to set progression criteria has been identified as a challenge in the literature.^64^ A 6‐month follow‐up time‐point was selected to examine the feasibility of longer‐term data collection. However, the study could have been strengthened by examining the feasibility of longer‐term follow‐up data collection e.g., 9–18 months post‐treatment. The majority of participants were female and may limit the generalizability of findings. Further, the majority of participants (78.7%) had an education level higher than upper secondary school, compared to 44% in the general Swedish population^65^ potentially further limiting generalizability. Our sample size was informed by recommendations primarily used for pilot RCTs^54^ and literature on informing sample sizes for single‐arm feasibility studies is lacking.^66^ There is a possibility study objectives could have been investigated with fewer participants. However, we examined the feasibility and acceptability of an internet‐administered intervention, which could be considered technically complex (e.g., including a range of technical elements such as a tab‐based interview view, film, audio files, in‐module exercises, online homework exercises, written messages via the Portal, and video‐conferencing), with a number of intervention components (e.g., four intervention modules and e‐therapist guidance). Literature suggests feasibility studies of interventions that are technically complex and include a number of components, may require a larger sample size than interventions with minimal complexity.^66^

### 4.2 Interpretation and implications for future research

While we successfully recruited our target sample size with an enrolment rate of 11.0%, confidence intervals ranged from 8.4% to 14.1% and in a future pilot RCT we will continue to identify participants via additional sources such as cancer organizations and interest groups. Further, we targeted parents of children treated for cancer with a self‐reported need for psychological support. Lack of recognition of one's psychological difficulties and lack of acknowledgement for the need of support, are commonly identified barriers to seeking help.^67^ Consequently, we may have failed to reach parents experiencing psychological difficulties who do not recognize or acknowledge a need for psychological support. Future research may look to identify methods to widen participation in the population and overcome potential barriers to help‐seeking, such as improving mental health literacy.^67^ However, it is important to note that of the 509 parents invited via the CCR, only 20% (*n* = 101) may be anticipated to experience at least mild symptoms of depression and/or anxiety.^6^ In depression trials utilizing recruitment strategies where study invitation letters are sent to patients identified via medical records with experience of depression, a recruitment rate of 12% may be anticipated.^48^, ^49^, ^51^ Given study invitations were sent to all parents identified via the CCR, rather than to parents with a known history of depression and/or anxiety, our enrolment rate of 11.0% may be considered as high. Despite overall recruitment success, we will strive for further improvements in the future pilot RCT, for example the use of personalized study invitation letters which resulted in improvement in recruitment rates, however small, in our embedded recruitment RCT^39^ with results reported separately.^40^ Future research may adopt similar strategies, including registry‐based recruitment^36^; an opt‐out recruitment strategy,^41^ and the use of personalized study invitation letters^40^ to optimize recruitment.

Our study dropout rate of 24.0% bettered progression criteria of ≤30%. Confidence intervals ranged from 14.9% to 35.3% and we aim to minimize study dropout in the forthcoming future pilot RCT by continuing to use retention strategies, including telephone reminders^59^ and study newsletters.^55^ In addition, assessment completion rates varied, with higher completion rates for the M.I.N.I. at each time‐point (84.4% at post‐treatment and 81.4% at follow‐up), in comparison to Portal assessment completion (65.6% at post‐treatment and 67.8% at follow‐up). Completion rates of weekly Portal assessments, to inform the process evaluation, were particularly low (decreasing over time from 65.7% to 38.9%). Difficulties with assessment completion are common.^68^ Less than satisfactory Portal assessment completion suggests in the future pilot RCT, we should minimize the number of online assessments used and seek to collect data over the telephone. For example, we will collect process evaluation data at three time‐points during the intervention over the telephone, rather than weekly via the Portal.

Our intervention adherence rate of 47.9% was slightly lower than progression criteria (≥50%) and there was no evidence of harm. Results suggest the intervention may be feasible and acceptable for the population and are in line with other research suggesting internet‐administered delivery mechanisms are acceptable to parents of children on cancer treatment^22^, ^23^ and parents of children previously treated for cancer.^25^, ^26^ Benefits of internet‐administered delivery may relate to flexibility of use and perceptions of privacy,^21^ overcoming common barriers to accessing support in the population such as guilt and putting the needs of the child before parents' own needs.^14^, ^15^ However, results also suggest a need to adapt the intervention to improve feasibility and acceptability before progressing to the future pilot RCT. While adherence to BA was high, adherence to WM was poor, especially for fathers. Challenges regarding adherence to internet‐administered interventions are common^69^, ^70^ and uptake within routine healthcare settings,^71^ including Sweden,^72^ is poor. Intervention acceptability is further explored in the embedded process evaluation, reported elsewhere, and will be used to adapt the intervention. However, adherence rates indicate a need to improve the acceptability of the intervention and there may be a need to improve the gender‐sensitivity of EJDeR, especially the WM module for fathers.

Recruitment of experienced research personnel was challenging^57^ delaying study set‐up. The use of paper‐based CRFs was time consuming and coordinating interviews with external personnel was resource intensive. The use of the TIC‐P (health economic outcome) was not feasible given the large amount of missing data. The Adult Service Use Schedule (AD‐SUS) developed from instruments used in similar trials^73^ will be used in the future pilot RCT.

Psychology program student e‐therapists did not have time to support caseloads and more experienced licensed psychologists and a CBT‐therapist supported the majority of participants. Further, psychology program student e‐therapists spent a mean time of 2.9 h per participant, per week, which is more therapist time than reported in other studies on guided internet‐administered CBT interventions.^74^, ^75^, ^76^ This finding may be explained by psychology program students only supporting a mean of 2.7 parents. Consequently, they may not have gained the opportunity to develop competence in using the support protocol and a clear understanding of the intervention structure and content, or how to use the Portal. Results indicate e‐therapist training and supervision should be improved (e.g. increase length of time for training, include role‐play, and revise training material) in future research to facilitate working with the intervention more efficiently.^77^ Additionally, recruiting part‐time employed e‐therapists could facilitate increased caseloads, potentially leading to increased efficiency.

In summary, the following modifications to the study protocol and EJDeR are warranted before commencing a pilot RCT: (1) collection of outcome assessment data via telephone; (2) reducing the number of measures; (3) adaptation of the intervention to improve the feasibility and acceptability of EJDeR; (4) recruitment of a trial coordinator; (5) recruitment of part‐time employed e‐therapists to increase caseloads and decrease time spent on each participant; (6) use of electronic CRFs to facilitate data collection and entry; and (7) training of research team members to collect research data over the telephone.

## 5 CONCLUSIONS

Using robust methods, including a priori specified progression criteria, the use of novel recruitment strategies^34^, ^40^, ^41^ and evidence‐based retention stragies,^55^, ^59^ our findings indicate methods, study procedures, and the intervention are feasible and acceptable and progression to a pilot RCT to prepare for the design and conduct of a future superiority RCT is warranted. The EJDeR intervention represents a promising and novel solution, delivered with minimal therapist guidance to meet parents' current unmet need for psychological support.

## AUTHOR CONTRIBUTIONS

**Ella Thiblin:** Data curation (equal); formal analysis (equal); investigation (equal); validation (equal); visualization (equal); writing – original draft (equal). **Joanne Woodford:** Formal analysis (equal); methodology (supporting); project administration (supporting); supervision (supporting); writing – original draft (equal). **Christina Reuther:** Data curation (equal); formal analysis (equal); investigation (equal); validation (equal); visualization (equal); writing – review and editing (supporting). **Johan Lundgren:** Supervision (supporting); writing – review and editing (supporting). **Nina Lutvica:** Data curation (supporting); investigation (equal); validation (supporting); writing – review and editing (supporting). **Louise von Essen:** Conceptualization (lead); funding acquisition (lead); methodology (lead); project administration (lead); resources (lead); supervision (lead); writing – review and editing (lead).

## FUNDING INFORMATION

This work is supported by the Swedish Research Council (grant number 521‐2014‐3337/E0333701, 2018‐02578, and 2021‐00868), the Swedish Cancer Society (grant number 15 0673 and 17 0709), the Swedish Childhood Cancer Foundation (grant number PR2017‐0005), and funding via the Swedish Research Council to U‐CARE, a Strategic Research environment (Dnr 2009‐1093). The funders had no role in study design, data collection and analysis, decision to publish, or preparation of the manuscript.

## CONFLICTS OF INTEREST

Declaration of interest: none.

## ETHICS APPROVAL STATEMENT

The ENGAGE feasibility trial was approved by the Regional Ethical Review Board in Uppsala, Sweden (Dnr: 2017/527) and was conducted in accordance with the Helsinki Declaration, ensuring the welfare and rights of all participants, and Good Clinical Practice (GCP) guidelines. Ethical amendment was obtained from Swedish Ethical Review Authority August 07, 2019, ref: 2019‐03083.

## PATIENT CONSENT STATEMENT

Not applicable.

## PERMISSION TO REPRODUCE MATERIAL FROM OTHER SOURCES

Not applicable.

## TRIAL REGISTRATION

ISRCTN 57233429.

## Supporting information


Data S1

## Data Availability

Data supporting feasibility objectives pertaining to recruitment and eligibility, data collection, attrition, and resources needed to complete the study and intervention are available in Zenodo at https://doi.org/10.5281/zenodo.6325611. Data stored in Zenodo supports Figure 1, Table 3 and Tables S2, S3, S7–S9. Access to the data stored in Zenodo is available upon written request from the corresponding author. Due to the nature of this research, participants of this study did not agree for their clinical data to be shared publicly, so supporting clinical data is not available and further ethical approval would be needed in order to share this data.
