# Supplementary material for: Internet‐administered, low‐intensity cognitive behavioral therapy for parents of children treated for cancer: A feasibility trial (ENGAGE)
Source: Cancer Med. 2022 Nov 20;12(5):6225–43. doi: 10.1002/cam4.5377 (PMC10028033; doi:10.1002/cam4.5377)
Supplement: Supplementary file 1 — Data S1 [file CAM4-12-6225-s001.zip › CAM4_5377_FigureS1.docx]

**Figure legends**

Figure 1: Study flow of participants in the ENGAGE feasibility trial. Note. Solid black lines denote participant flow through the study, including study drop outs i.e., those who discontinued the study. Dashed grey lines represent participants that were lost to follow-up during assessments at post-treatment (12 weeks) and follow-up (6 months) respectively, but had not dropped out of the study.

Supporting Figure 1: An overview of EJDeR. © Joanne Woodford, Paul Farrand, Josefin Hagström, Li Hedenmalm, Louise von Essen. Originally published in JMIR Formative Research (<https://formative.jmir.ord>), 22.07.2021. This is an open-access article distributed under the terms of the Creative Commons Attribution License (<https://creativecommons.org/licenses/by/4.0>), which permits unrestricted use, distribution and reproduction in any medium, provided the original work, first published in JMIR Formative Research, is properly cited. The complete bibliographic information, a link to the original publication on <https://jmir.org>, as well as this copyright and license information must be included.
