# Supplementary material for: Internet‐administered, low‐intensity cognitive behavioral therapy for parents of children treated for cancer: A feasibility trial (ENGAGE)
Source: Cancer Med. 2022 Nov 20;12(5):6225–43. doi: 10.1002/cam4.5377 (PMC10028033; doi:10.1002/cam4.5377)
Supplement: Supplementary file 1 — Data S1 [file CAM4-12-6225-s001.zip › CAM4_5377_Supporting tables.docx]

**Supporting tables**

Supporting table 1. Original versus revised feasibility outcomes and progression criteria

Supporting table 2. Reasons for non-participation in response to the closed multiple-choice question (N = 137)

Supporting table 3. Number and percentages of participants completing weekly Portal assessments of the total sample size at each week of the intervention

Supporting table 4. Missing items in the M.I.N.I., by specific modules and by total items, at eligibility, post-treatment, and follow-up

Supporting table 5. Missing items in Portal assessment, by specific measures and by total items, at baseline, post-treatment, and follow-up

Supporting table 6. Missing items in weekly Portal assessments, by specific measures and by total items, each week of the intervention

Supporting table 7. Descriptive statistics for length of time required for participants to work through EJDeR, and to complete assessments at baseline, post-treatment, and follow-up

Supporting table 8. Descriptive statistics for reminder contacts during recruitment and to complete Portal assessments at recruitment, baseline, post-treatment, and follow-up

Supporting table 9. Descriptive statistics for contacts needed to arrange eligibility interview, M.I.N.I. and semi-structured interview at eligibility, baseline, post-treatment, and follow-up

Supporting table 10. Internet usage for participants (N = 75)

Supporting table 11. Baseline sociodemographic and clinical characteristics for children treated for cancer

Supporting table 12. Participants meeting criteria for diagnosis in accordance with the M.I.N.I. at baseline, post-treatment, and follow-up

Supporting table 13. Treatment Inventory of Costs in Patients with psychiatric disorders (TIC-P) at baseline and post-treatment

**Supporting table 1.** Original versus revised feasibility outcomes and progression criteria

| **Outcome** | **Original protocol evaluation** | **Revised protocol evaluation** | **Original progression criteria to controlled trial** | **Changes to progression criteria to controlled trial^a^** |
| --- | --- | --- | --- | --- |
| Recruitment and eligibility | Number identified via the Swedish Childhood Cancer Registry and the Swedish Tax Agency and/or via advertisements | Number identified via postal study invitations (Swedish Childhood Cancer Registry and the Swedish Tax Agency [NAVET]) and/or via Online advertisements via cancer organizations and interest groups | No criteria set | - |
|  | Percentage assessed for eligibility; fulfilling inclusion criteria, and included (of total number identified) | Percentage consented to participate, assessed for eligibility, fulfilling eligibility criteria, and enrolled (of total number invited) | ≥9% enrolled of total participant population invited (e.g., interested in participating of total participant population invited) | ≥9% enrolled of total participant population invited (e.g., included of total participant population invited) |
|  | Reasons for ineligibility | Reasons for ineligibility | No criteria set | - |
|  | Ambiguities regarding eligibility criteria | Ambiguities regarding eligibility criteria including diagnostic uncertainties in M.I.N.I. | No criteria set | - |
|  | Reasons for non-participation | Reasons for non-participation | No criteria set | - |
| Data collection | Percentage completing assessments | Percentage completing assessments  M.I.N.I. (eligibility interview, post-treatment and, follow-up)  Semi-structured interview (baseline and post-treatment)  Portal assessment (baseline, post-treatment, and follow-up)  Weekly Portal assessment | ≥70% answering all questions at all assessments | - |
|  | Numbers of missing items | Numbers of missing items  M.I.N.I. (eligibility interview, post-treatment, and follow-up)  Portal assessment (baseline, post-treatment, and follow-up)  Weekly Portal assessment | ≤10% per questionnaire | ≤10% per measure |
|  | Types and number of potential uncertainties in diagnostic interviews | ~~Types and number of potential uncertainties in diagnostic interviews~~^a^ | No criteria set | ~~-~~ |
| Attrition | Rates of study dropout Rate of intervention dropout | Rate of study dropout  Rate of intervention dropout | ≤30%  ≤30% | - |
| Resources needed to complete the study and the intervention | Length of time required for:  Participants to work through the intervention  Participants to complete questionnaires and interviews  E-therapists to deliver the intervention  Study personnel to administer the study | Length of time required for:  Participants to work through the intervention  Participants to complete the initial assessment session and mid-intervention booster session with e-therapist  Participants to complete the eligibility interview, M.I.N.I., semi-structured interview, Portal assessment at each time-point  E-therapists to deliver the intervention  ~~Study personnel to administer the study~~^b^ | No criteria set | - |
|  |  | Number of:  Internal and external study personnel  Reminder contacts needed during recruitment  Reminder contacts needed to complete Portal assessment at each time-point  Contacts needed to arrange eligibility interview, M.I.N.I. and semi-structured interview over the telephone at each time-point | No criteria set | - |
| Participants’ adherence to intervention | Number of:  Opened introductory chapters  Opened CBT modules, completed action plans  Completed video or telephone assessment sessions  Completed ‘booster’ support sessions | Number of:  Participants adhering to the minimum treatment dose (MTD)  Opened modules  Completed LICBT modules started with  Completed initial assessment session  Completed mid-intervention booster session  Completed homework sheets | ≥50% attending the video or telephone initial assessment session, completing the introductory chapter, 5 CBT modules and the ‘booster’ support session | ≥50% adhering to minimum treatment dose (MTD), ie. attending the initial assessment session, completing the introduction and psychoeducation module and one LICBT treatment module (ie. behavioral activation or worry management) and attending the mid-intervention booster session. |
| Participants’ use of the intervention | Number of:  Logins  Use of optional support functions | Number of:  Participant logins  Participant written messages  E-therapist written messages  ~~Use of optional support functions~~^c^ | No criteria set | ~~-~~ |
| E-therapists’ adherence to intervention | Content of internet-administered written e-therapist-parent communication | Content of initial assessment, mid-intervention booster, and written messages via the Portal | No criteria set | - |
| Participants’ acceptability of the intervention and data collection ~~and exploration of mechanisms of impact~~ | Reasons for poor attendance and withdrawal from study and intervention  Impressions and experiences of working with the intervention (including positive and negative consequences) and of completing questionnaires and interviews | Reasons for low adherence and dropout from study and intervention  Number of risk assessments  Impressions and experiences of working with the intervention (including positive and negative consequences) and of completing assessments and interviews | No criteria set  No criteria set  ≥70% of participants using the intervention reporting that it is helpful  <1 participant reporting substantial negative consequences related to participation in the study and/or intervention | -  ~~≥70% of participants using the intervention reporting that it is helpful~~^d^ |

Protocol modifications and minor revisions to the protocol to improve clarity are highlighted to improve transparency.

Feasibility outcomes that were not collected due to protocol modifications are depicted with a horizontal line through their centre.

^a^Feasibility outcome was deleted as outcome is addressed by “Ambiguities regarding eligibility criteria”.

^b^Feasibility outcome was deleted as it was not possible to calculate the length of time taken for study personnel to administer the study. However, the number of internal and external study personnel, reminder contacts, and contacts need to arrange the eligibility interview, M.I.N.I. interview and semi-structured interviews over the telephone at each time-point and reported as proxy estimates.

^c^Feasibility outcome deleted as optional support functions were not used in the final version of the EJDeR intervention.

^d^Feasibility outcome deleted as a question concerning whether participants using the intervention reported it as helpful was omitted due to researcher error.

**Supporting table 2.** Reasons for non-participation in response to the closed multiple-choice question (N = 137)

| **Reasons** | **Total** | |
| --- | --- | --- |
|  | **n** | **%** |
| I do not experience any need for psychological support | 93 | 67.9 |
| I do not have enough time | 53 | 38.7 |
| I am not interested in using internet-administered self-help | 23 | 16.8 |
| I am too tired to participate in a research project | 20 | 14.6 |
| I do not want to participate in a research project | 17 | 12.4 |
| I do not like talking about my personal problems | 11 | 8.0 |
| My physical health is hindering me | 4 | 2.9 |
| I do not want to participate because of my child’s health condition | 2 | 1.5 |
| I do not want to participate because my child recently died | 1 | 0.7 |

Multiple responses possible.

**Supporting table 3.** Number and percentages of participants completing weekly Portal assessment of the total sample size at each week of the intervention

| **Week number** | **N** | **Completed assessment^a^** | | |
| --- | --- | --- | --- | --- |
|  |  | **n** | **%** | **95% CI** |
| Week 1 | 70 | 46 | 65.7 | 53.4, 76.6 |
| Week 2 | 68 | 39 | 57.4 | 44.8, 69.3 |
| Week 3 | 64 | 38 | 59.4 | 46.4, 71.5 |
| Week 4 | 61 | 34 | 55.7 | 42.5, 68.5 |
| Week 5 | 58 | 33 | 56.9 | 43.2, 69.8 |
| Week 6 | 58 | 36 | 62.1 | 48.4, 74.5 |
| Week 7 | 57 | 27 | 47.4 | 33.9, 61.0 |
| Week 8 | 56 | 24 | 42.9 | 29.7, 56.8 |
| Week 9 | 55 | 25 | 45.5 | 32.0, 59.5 |
| Week 10 | 55 | 22 | 40.0 | 27.0, 54.1 |
| Week 11 | 54 | 21 | 38.9 | 25.9, 53.1 |

N = the total sample size at each week of the intervention (e.g., the number of participants that remained in the intervention at each time-point).

^a^Completed assessment defined as no more than two measures missing in total out of the five measures included within each weekly Portal assessment.

**Supporting table 4.** Missing items in the M.I.N.I., by specific modules and by total items, at eligibility, post-treatment, and follow-up

| **M.I.N.I. module** | **Eligibility (n = 75)** | | | | **Post-treatment (n = 54 )** | | | | **Follow-up (n = 48)** | | | |
| --- | --- | --- | --- | --- | --- | --- | --- | --- | --- | --- | --- | --- |
|  | **Total items, N** | **Missing, n** | **Missing, %** | **95% CI^a^** | **Total items, N** | **Missing,n** | **Missing, %** | **95% CI^a^** | **Total items, N** | **Missing, n** | **Missing, %** | **95% CI^a^** |
| MDD | 2100 | 97 | 4.6 | 3.8, 5.6 | 1512 | 27 | 1.8 | 1.2, 2.6 | 1344 | 27 | 2.0 | 1.3, 2.9 |
| PD | 1650 | 10 | 0.6 | 0.3, 1.1 | 1188 | 2 | 0.2 | 0.0, 0.6 | 1056 | 4 | 0.4 | 0.1, 1.0 |
| Agoraphobia | 600 | 1 | 0.2 | 0.0, 0.9 | 432 | 2 | 0.5 | 0.1, 1.7 | 384 | 0 | 0.0 | 0.0, 1.0 |
| SAD | 600 | 2 | 0.3 | 0.0, 1.2 | 432 | 0 | 0.0 | 0.0, 0.9 | 384 | 9 | 2.3 | 1.1, 4.4 |
| OCD | 675 | 11 | 1.6 | 0.8, 2.9 | 486 | 0 | 0.0 | 0.0, 0.8 | 432 | 9 | 2.1 | 1.0, 3.9 |
| PTSD | 1875 | 1 | 0.1 | 0.0, 0.3 | 1350 | 0 | 0.0 | 0.0, 0.3 | 1200 | 0 | 0.0 | 0.0, 0.3 |
| AUD | 1950 | 55 | 2.8 | 2.1, 3.7 | 1404 | 28 | 2.0 | 1.3, 2.9 | 1248 | 20 | 1.6 | 1.0, 2.5 |
| SUD | 1350 | 0 | 0.0 | 0.0, 0.3 | 972 | 2 | 0.2 | 0.0, 0.7 | 864 | 0 | 0.0 | 0.0, 0.4 |
| GAD | 900 | 45 | 5.0 | 3.7, 6.6 | 648 | 3 | 0.5 | 0.1, 1.4 | 576 | 3 | 0.5 | 0.1, 1.5 |
| Suicidality | 3525 | 377 | 10.7 | 9.7, 11.8 | 2538 | 98 | 3.9 | 3.2, 4.7 | 2256 | 28 | 1.2 | 0.8, 1.8 |
| **M.I.N.I. total items** | 15225 | 599 | 3.9 | 3.6, 4.3 | 10962 | 162 | 1.5 | 1.3, 1.7 | 9744 | 100 | 1.0 | 0.8, 1.3 |

Abbreviations: AUD, Alcohol Use Disorder; GAD, Generalized Anxiety Disorder; MDD, Major Depressive Disorder; M.I.N.I., Mini-International Neuropsychiatric

Interview version 7.0.0; OCD, Obsessive-Compulsive Disorder; PD, Panic Disorder; PTSD, Post-traumatic Stress Disorder; SAD, Social Anxiety Disorder; SUD, Substance

Use Disorder (Non-Alcohol).

^a^CIs calculated from 0 are one-sided 97.5% CIs.

**Supporting table 5.** Missing items in Portal assessment, by specific measures and by total items, at baseline, post-treatment, and follow-up

| **Measure** | **Baseline** | | | | | **Post-treatment** | | | | | **Follow-up** | | | | |
| --- | --- | --- | --- | --- | --- | --- | --- | --- | --- | --- | --- | --- | --- | --- | --- |
|  | **Sample** | **Total items** | **Missing items** | **Missing items** | **95% CI^a^** | **Sample** | **Total items** | **Missing items** | **Missing items** | **95% CI^a^** | **Sample** | **Total items** | **Missing items** | **Missing items** | **95% CI^a^** |
|  | **N** | **N** | **n** | **%** |  | **N** | **N** | **n** | **%** |  | **N** | **N** | **n** | **%** |  |
| PCL-5 | 72 | 1440 | 0 | 0.0 | 0.0, 0.3 | 42 | 840 | 2 | 0.2 | 0.0, 0.9 | 40 | 800 | 1 | 0.1 | 0.0, 0.7 |
| PCL-C | 72 | 1224 | 0 | 0.0 | 0.0, 0.3 | 42 | 714 | 0 | 0.0 | 0.0, 0.5 | 40 | 680 | 0 | 0.0 | 0.0, 0.5 |
| PHQ-9 | 72 | 648 | 1^b^ | 0.2 | 0.0, 0.9 | 42 | 378 | 0 | 0.0 | 0.0, 1.0 | 40 | 360 | 0 | 0.0 | 0.0, 1.0 |
| GAD-7 | 72 | 504 | 0 | 0.0 | 0.0, 0.7 | 42 | 294 | 0 | 0.0 | 0.0, 1.3 | 40 | 280 | 0 | 0.0 | 0.0, 1.3 |
| FRHC | 72 | 144 | 0 | 0.0 | 0.0, 2.5 | 42 | 84 | 0 | 0.0 | 0.0, 4.3 | 40 | 80 | 0 | 0.0 | 0.0, 4.5 |
| AAQ-6 | 72 | 432 | 0 | 0.0 | 0.0, 0.9 | 42 | 252 | 6 | 2.4 | 0.9, 5.1 | 40 | 240 | 0 | 0.0 | 0.0, 1.5 |
| BADS | 72 | 1800 | 0 | 0.0 | 0.0, 0.2 | 42 | 1050 | 2 | 0.2 | 0.0, 0.7 | 40 | 1000 | 0 | 0.0 | 0.0, 0.4 |
| FSS | 72 | 648 | 0 | 0.0 | 0.0, 0.6 | 42 | 378 | 1 | 0.3 | 0.0, 1.5 | 40 | 360 | 0 | 0.0 | 0.0, 1.0 |
| EQ-5D | 72 | 432 | 0 | 0.0 | 0.0, 0.9 | 42 | 252 | 0 | 0.0 | 0.0, 1.5 | 40 | 240 | 0 | 0.0 | 0.0, 1.5 |
| SCS-SF | 72 | 864 | 0 | 0.0 | 0.0, 0.4 | 42 | 504 | 1 | 0.2 | 0.0, 1.1 | 40 | 480 | 1 | 0.2 | 0.0, 1.2 |
| **Total items** |  | 8136 | 1 | 0.0 | 0.0, 0.1 |  | 4746 | 12 | 0.3 | 0.1, 0.4 |  | 4520 | 2 | 0.0 | 0.0, 0.2 |

Abbreviations: AAQ-6, Acceptance and Action Questionnaire; BADS, Behavioral Activation for Depression Scale; EQ-5D, EuroQol 5-dimension questionnaire, including visual analogue scale; FSS, Fatigue Severity Scale; FRHC, Fear of recurrence and serious health condition (structured questions); GAD-7, Generalized Anxiety Disorder 7-item scale; PCL-5, Post-traumatic Stress Disorder Checklist for DSM-5; PCL-C, adapted version of Post-traumatic Stress Disorder Checklist-Civilian version; PHQ-9, Patient Health Questionnaire; PTSS, Post-traumatic Stress Symptoms; SCS-SF, Self-Compassion Scale-Short Form.

Sample, N refers to the total number of participants who completed the Portal assessment at each time-point.

^a^CIs calculated from 0 are one-sided 97.5% CIs.

^b^Item missing due to researcher error (Portal assessment conducted over the telephone and item missed).

**Supporting table 6.** Missing items in weekly Portal assessment, by specific measures and by total items, each week of the intervention

| **Measure** | **Sample, N** | **Total items, N** | **Missing items, n** | | **Missing items, %** | | **95% CI^a^** |
| --- | --- | --- | --- | --- | --- | --- | --- |
| **Week 1** | | | | | | | |
| PCL-5 | 48 | 960 | 40 | 4.2 | | 3.0, 5.6 | |
| PCL-C | 48 | 816 | 34 | 4.2 | | 2.9, 5.8 | |
| PHQ-9 | 48 | 432 | 18 | 4.2 | | 2.5, 6.5 | |
| AAQ-6 | 48 | 288 | 12 | 4.2 | | 2.2, 7.2 | |
| BADS | 48 | 1200 | 0 | 0.0 | | 0.0, 0.3 | |
| Total |  | 3696 | 104 | 2.8 | | 2.3, 3.4 | |
| **Week 2** | | | | | | | |
| PCL-5 | 40 | 800 | 21 | 2.6 | | 1.6, 4.0 | |
| PCL-C | 40 | 680 | 17 | 2.5 | | 1.5, 4.0 | |
| PHQ-9 | 40 | 360 | 9 | 2.5 | | 1.2, 4.7 | |
| AAQ-6 | 40 | 240 | 6 | 2.5 | | 0.9, 5.4 | |
| BADS | 40 | 1000 | 0 | 0.0 | | 0.0, 0.4 | |
| Total |  | 3080 | 53 | 1.7 | | 1.3, 2.2 | |
| **Week 3** | | | | | | | |
| PCL-5 | 38 | 760 | 0 | 0.0 | | 0.0, 0.5 | |
| PCL-C | 38 | 646 | 0 | 0.0 | | 0.0, 0.6 | |
| PHQ-9 | 38 | 342 | 0 | 0.0 | | 0.0, 1.1 | |
| AAQ-6 | 38 | 228 | 0 | 0.0 | | 0.0, 1.6 | |
| BADS | 38 | 950 | 0 | 0.0 | | 0.0, 0.4 | |
| Total |  | 2926 | 0 | 0.0 | | 0.0, 0.1 | |
| **Week 4** | | | | | | | |
| PCL-5 | 34 | 680 | 0 | 0.0 | | 0.0, 0.5 | |
| PCL-C | 34 | 578 | 0 | 0.0 | | 0.0, 0.6 | |
| PHQ-9 | 34 | 306 | 0 | 0.0 | | 0.0, 1.2 | |
| AAQ-6 | 34 | 204 | 0 | 0.0 | | 0.0, 1.8 | |
| BADS | 34 | 850 | 2 | 0.2 | | 0.0, 0.9 | |
| Total |  | 2618 | 2 | 0.0 | | 0.0, 0.3 | |
| **Week 5** | | | | | | | |
| PCL-5 | 35 | 700 | 41 | 5.9 | | 4.2, 7.9 | |
| PCL-C | 35 | 595 | 35 | 5.9 | | 4.1, 8.1 | |
| PHQ-9 | 35 | 315 | 18 | 5.7 | | 3.4, 8.9 | |
| AAQ-6 | 35 | 210 | 12 | 5.7 | | 3.0, 9.8 | |
| BADS | 35 | 875 | 4 | 0.5 | | 0.1, 1.2 | |
| Total |  | 2695 | 110 | 4.1 | | 3.4, 4.9 | |
| **Week 6** | | | | | | | |
| PCL-5 | 36 | 720 | 1 | 0.1 | | 0.0, 0.8 | |
| PCL-C | 36 | 612 | 2 | 0.3 | | 0.0, 1.2 | |
| PHQ-9 | 36 | 324 | 0 | 0.0 | | 0.0, 1.1 | |
| AAQ-6 | 36 | 216 | 0 | 0.0 | | 0.0, 1.7 | |
| BADS | 36 | 900 | 2 | 0.2 | | 0.0, 0.8 | |
| Total |  | 2772 | 5 | 0.2 | | 0.1, 0.4 | |
| **Week 7** | | | | | | | |
| PCL-5 | 27 | 540 | 0 | 0.0 | | 0.0, 0.7 | |
| PCL-C | 27 | 459 | 0 | 0.0 | | 0.0, 0.8 | |
| PHQ-9 | 27 | 243 | 0 | 0.0 | | 0.0, 1.5 | |
| AAQ-6 | 27 | 162 | 0 | 0.0 | | 0.0, 2.3 | |
| BADS | 27 | 675 | 0 | 0.0 | | 0.0, 0.6 | |
| Total |  | 2079 | 0 | 0.0 | | 0.0, 0.2 | |
| **Week 8** | | | | | | | |
| PCL-5 | 25 | 500 | 20 | 4.0 | | 2.5, 6.1 | |
| PCL-C | 25 | 425 | 17 | 4.0 | | 2.4, 6.3 | |
| PHQ-9 | 25 | 225 | 0 | 0.0 | | 0.0, 1.6 | |
| AAQ-6 | 25 | 150 | 6 | 4.0 | | 1.5, 8.5 | |
| BADS | 25 | 625 | 0 | 0.0 | | 0.0, 0.6 | |
| Total |  | 1925 | 43 | 2.2 | | 1.6, 3.0 | |
| **Week 9** | | | | | | | |
| PCL-5 | 26 | 520 | 21 | 4.0 | | 2.5, 6.1 | |
| PCL-C | 26 | 442 | 18 | 4.1 | | 2.4, 6.4 | |
| PHQ-9 | 26 | 234 | 9 | 3.9 | | 1.8, 7.2 | |
| AAQ-6 | 26 | 156 | 6 | 3.9 | | 1.4, 8.2 | |
| BADS | 26 | 650 | 3 | 0.5 | | 0.1, 1.3 | |
| Total |  | 2002 | 57 | 2.9 | | 2.2, 3.7 | |
| **Week 10** |  |  |  |  | |  | |
| PCL-5 | 22 | 440 | 2 | 0.5 | | 0.1, 1.6 | |
| PCL-C | 22 | 374 | 2 | 0.5 | | 0.1, 1.9 | |
| PHQ-9 | 22 | 198 | 0 | 0.0 | | 0.0, 1.9 | |
| AAQ-6 | 22 | 132 | 6 | 4.6 | | 1.7, 9.6 | |
| BADS | 22 | 550 | 0 | 0.0 | | 0.0, 0.7 | |
| Total |  | 1694 | 10 | 0.6 | | 0.3, 1.1 | |
| **Week 11** | | | | | | | |
| PCL-5 | 21 | 420 | 0 | 0.0 | | 0.0, 0.9 | |
| PCL-C | 21 | 357 | 0 | 0.0 | | 0.0, 1.0 | |
| PHQ-9 | 21 | 189 | 0 | 0.0 | | 0.0, 1.9 | |
| AAQ-6 | 21 | 126 | 0 | 0.0 | | 0.0, 2.9 | |
| BADS | 21 | 525 | 0 | 0.0 | | 0.0, 0.7 | |
| Total |  | 1617 | 0 | 0.0 | | 0.0, 0.2 | |

Abbreviations: AAQ-6, Acceptance and Action Questionnaire; BADS, Behavioral Activation for Depression Scale; PCL-5, Post-traumatic Stress Disorder Checklist for DSM-5; PCL-C, adapted version of Post- traumatic Stress Disorder Checklist-Civilian version; PHQ-9, Patient Health Questionnaire.

Sample, N refers to the total number of participants who started the weekly Portal assessment each week.

^a^CIs calculated from 0 are one-sided 97.5% CIs.

**Supporting table 7**. Descriptive statistics for length of time required for participants to work through EJDeR, and to complete assessments at baseline, post-treatment, and follow-up

|  | **Length of time** | | | | |  |
| --- | --- | --- | --- | --- | --- | --- |
| **EJDeR** | **N** | **n** | **M** | **SD** | **Min** | **Max** |
| Time participants had access to EJDeR (days) | 72 | 72 | 73.2 | 32.1 | 0.0 | 126.0 |
| Time spent on EJDeR on the Portal (minutes) | 72 | 72 | 205.1 | 153.6 | 8.6 | 611.7 |
| Time to complete the initial assessment session (minutes) | 62 | 57^a^ | 45.3 | 8.3 | 26.1 | 64.5 |
| Time to complete the mid-intervention booster session (minutes) | 44 | 41^b^ | 40.2 | 9.5 | 18.3 | 60.1 |
| **Assessment time-points** | **N** | **n** | **M** | **SD** | **Min** | **Max** |
| Eligibility | | | | | |  |
| Time to complete eligibility interview (minutes) | 76 | 73^c^ | 40.2 | 10.3 | 20.0 | 68.0 |
| Baseline | | | | | |  |
| Time to complete semi-structured interview (minutes) | 74 | 73^d^ | 37.7 | 10.8 | 17.0 | 67.0 |
| Time to complete Portal assessment (minutes) | 72 | 60^e^ | 29.5 | 13.5 | 12.1 | 76.9 |
| Post-treatment | | | | | |  |
| Time to complete M.I.N.I. (minutes) | 54 | 49^f^ | 19.3 | 6.7 | 10.0 | 41.0 |
| Time to complete semi-structured interview (minutes) | 53 | 53 | 28.7 | 8.2 | 14.0 | 46.0 |
| Time to complete Portal assessment (minutes) | 42 | 39^g^ | 18.7 | 7.9 | 7.9 | 46.9 |
| Follow-up | | | | | |  |
| Time to complete M.I.N.I. (minutes) | 48 | 47^h^ | 18.5 | 5.6 | 9.0 | 30.0 |
| Time to complete Portal assessment (minutes) | 40 | 35^i^ | 20.1 | 6.0 | 10.9 | 36.2 |

N = total number of participants using EJDeR and completing assessments at each time-point.

n = total number of participants with length of time was successfully audio recorded or logged on the Portal. Length of time was rounded to the nearest minute when recorded using external audio recorders (e.g., length of time to complete interviews at each assessment time-point).

Length of time was logged to the second on the Portal.

^a^n = 5/62 initial assessment sessions not recorded (technical error: n = 1; e-therapist error: n = 3; no consent: n = 1)

^b^n = 3/44 mid-intervention booster sessions not recorded (technical error: n = 1; e-therapist error: n = 1; no consent, n = 1)

^c^n = 3/76 eligibility interviews not recorded (technical error: n = 1; no consent, n = 2)

^d^n = 1/74 semi-structured interview at baseline not recorded (technical error: n = 1)

^e^n = 12/72 Portal assessments at baseline not logged (completed over the telephone: n = 3; did Portal assessments over more than one sitting and not possible to calculate time to complete, n = 9)

^f^n = 5/54 M.I.N.I. at post-treatment not recorded (technical error: n = 4; no consent: n = 1)

^g^n = 3/42 Portal assessments at post-treatment not logged (completed over the telephone: n = 2; did Portal assessments over more than one sitting, not possible to calculate time to complete, n = 1)

^h^n = 1/48 M.I.N.I. at follow-up not recorded (no consent: n = 1)

^i^n = 5/40 Portal assessments at follow-up not logged (completed over the telephone: n = 2; did Portal assessments over more than one sitting, not possible to calculate time to complete, n = 3)

**Supporting table 8**. Descriptive statistics for reminder contacts during recruitment and to complete Portal assessments at recruitment, baseline, post-treatment, and follow-up

| **Time-point** | **N** | **Mean** | **SD** | **Min** | **Max** |
| --- | --- | --- | --- | --- | --- |
| Recruitment |  |  |  |  |  |
| Two weeks after registering interest in participation | 12 | 2.8 | 1.9 | 1 | 6 |
| Four weeks after receiving a study invitation | 215 | 3.6 | 2.2 | 1 | 13 |
| Reminder contacts to complete Portal assessments: |  |  |  |  |  |
| Baseline | 10 | 1.6 | 1.0 | 1 | 4 |
| Post-treatment | 28 | 3.1 | 2.2 | 1 | 8 |
| Follow-up | 28 | 4.4 | 2.1 | 1 | 8 |

N = the total number of participants who received at least one reminder contact.

Contacts are defined as any contact (successful or unsuccessful attempts) via telephone, SMS or e-mail

made by either a member of the research team or made by participants to contact the research team in

response to reminders.

**Supporting table 9**. Descriptive statistics for contacts needed to arrange eligibility interview, M.I.N.I. and semi-structured interview at eligibility, baseline, post-treatment, and follow-up

| **Interview** | **N** | **Mean** | **SD** | **Min** | **Max** |
| --- | --- | --- | --- | --- | --- |
| Eligibility interview | 81^a^ | 4.8 | 2.3 | 2 | 14 |
| Semi-structured interview at baseline | 75 | 3.4 | 2.6 | 1 | 16 |
| M.I.N.I. and semi-structured interview at post-treatment | 65^b^ | 5.4 | 2.9 | 1 | 15 |
| M.I.N.I. at follow-up | 59 | 6.9 | 3.7 | 3 | 18 |

N = the total number of participants who received at least one contact at each time-point (at baseline, post-treatment, and follow-up).

Contacts are defined as any contact attempt (successful or unsuccessful) via telephone, SMS, or e-mail,

made by either a member of the research team and/or participants.

^a^N = 81 potential participants consented to participant and contact attempts were made to arrange eligibility interviews.

^b^One participant was contacted by mistake who had dropped out of the study.

**Supporting table 10**. Internet usage for participants (N = 75)

| **Internet usage** | **n (%)** | | | |
| --- | --- | --- | --- | --- |
| Length of time of daily use | | | | |
| <1 hour | 0 (0.0) | | | |
| 1-2 hours | 26 (34.7) | | | |
| 3-5 hours | 30 (40.0) | | | |
| >5 hours | 19 (25.3) | | | |
| Type of internet connection^a^ | | | | |
| Broadband | 71 (94.7) | | | |
| Modem | 5 (6.7) | | | |
| Do not know | 1 (1.3) | | | |
| Frequency of using the internet for different activities | Often | Sometimes | Never | Missing |
| E-mail | 66 (88.0) | 9 (12.0) | 0 (0.0) | 0 (0.0) |
| Information | 62 (82.7) | 12 (16.0) | 1 (1.3) | 0 (0.0) |
| Work/business/studies | 58 (77.3) | 13 (17.3) | 4 (5.3) | 0 (0.0) |
| Chat/groups on social media/forums chat/forums | 37 (49.3) | 31 (41.3) | 6 (8.0) | 1 (1.3) |
| Downloading media (e.g., music, movies) | 28 (37.3) | 30 (40.0) | 17 (22.7) | 0 (0.0) |
| Shopping/auctions | 15 (20.0) | 54 (72.0) | 6 (8.0) | 0 (0.0) |
| Games | 7 (9.3) | 26 (34.7) | 41 (54.7) | 1 (1.3) |
| Other activities | 3 (4.0) | 13 (17.3) | 16 (21.3) | 43 (57.3) |

Data are number (%).

Percentages may not always total 100 due to rounding.

^a^Selecting both broadband and modem was possible.

**Supporting table 11**. Baseline sociodemographic and clinical characteristics for children treated for cancer

| **Sociodemographic and clinical characteristics** | **Swedish Childhood Cancer Registry data (N = 65)^a^** | **Parent self-report data**  **(N = 70)^b^** |
| --- | --- | --- |
| Current age (years) |  |  |
| Mean (SD) range | 10.6 (5.0) 4–24 | 10.6 (5.2) 2–24 |
| Gender |  |  |
| Female | 31 (47.7) | 32 (45.7) |
| Male | 34 (52.3) | 38 (54.3) |
| Cancer diagnosis |  |  |
| Blastoma | 13 (20.0) | 12 (17.1) |
| Carcinoma | 1 (1.5) | 1 (1.4) |
| CNS tumor | 7 (10.8) | 8 (11.4) |
| Germ cell tumor | 1 (1.5) | 2 (2.9) |
| Leukemia | 31 (47.7) | 32 (45.7) |
| Lymphoma | 9 (13.8) | 10 (14.3) |
| Sarcoma | 3 (4.6) | 4 (5.7) |
| Do not know | 0 (0.0) | 1(1.4) |
| Time since first diagnosis (years) |  |  |
| ≥1 – <2 | 2 (3.1) | - |
| ≥2 – <3 | 7 (10.8) | - |
| ≥3 – <4 | 15 (23.1) | - |
| ≥4 – <5 | 16 (24.6) | - |
| ≥5 – <6 | 11 (16.9) | - |
| ≥6 – <7 | 10 (15.4) | - |
| ≥7 – <8 | 4 (6.2) | - |
| Time since end of treatment (years) |  |  |
| >1 | 10 (15.4) | 7 (10.0) |
| ≥1 – <2 | 13 (20.0) | 8 (11.4) |
| ≥2 – <3 | 10 (15.4) | 2 (2.9) |
| ≥3 – <4 | 12 (18.5) | 2 (2.9) |
| ≥4 – <5 | 10 (15.4) | 1 (1.4) |
| ≥5 – <6 | 10 (15.4) | 2 (2.9) |
| Missing | 0 (0.0) | 48 (68.6) |
| Type of cancer treatment^c^ |  |  |
| Chemotherapy | 21 (32.3) | 55 (78.6) |
| Surgery | 4 (6.2) | 25 (35.7) |
| Radiation Therapy | 9 (13.8) | 14 (20.0) |
| Stem Cell or Bone Marrow Transplant | 0 (0.0) | 5 (7.1) |
| Other | 0 (0.0) | 1 (1.4) |
| Missing | 40 (61.5) | 10 (14.3) |
| Cancer recurrence |  |  |
| Yes | - | 5 (7.1) |
| No | - | 65 (92.9) |

Data are number (%) unless stated otherwise.

Percentages may not always total 100 due to rounding.

Both data from the Swedish Childhood Cancer Registry and from parents (self-report) was collected as the Swedish Childhood Cancer Registry is not updated in real-time and therefore the data provided by the Swedish Childhood Cancer Registry may not have been completely up-to-date at the time participants were recruited into the study.

^a^Data for n = 5 children treated for cancer whose parents were recruited via online advertisements via cancer organizations and interest groups was not available from the Swedish Childhood Cancer Registry, data for n = 1 child was deleted in error when the parent dropped out of the study. Data for children with both parents participating in the study (n = 4) are only included once.

^b^Data for children with both parents participating in the study, out of the whole study sample (n = 5), are only included once.

^c^Multiple responses possible.

**Supporting table 12.** Participants meeting criteria for diagnosis in accordance with the M.I.N.I. at baseline, post-treatment, and follow-up

| **Diagnosis** | **Baseline**  **(N = 75)** | **Post-treatment (N = 54)** | **Follow-up (N = 48)** |
| --- | --- | --- | --- |
|  | **n (%)** | **n (%)** | **n (%)** |
| Meeting criteria for diagnosis for any disorder | 45 (60.0) | 31 (57.4) | 20 (41.7) |
| Major Depressive Disorder |  |  |  |
| Current (2 Weeks) | 5 (6.7) | 1 (1.9) | 1 (2.1) |
| Past | 39 (52.0) | 27 (50.0) | 20 (41.7) |
| Recurrent^a^ | 14 (18.7) | 15 (27.8) | 15 (31.3) |
| Panic Disorder |  |  |  |
| Current (2 Weeks) | 2 (2.7) | 0 (0.0) | 1 (2.1) |
| Lifetime^b^ | 11 (14.7) | 10 (18.5) | 7 (14.6) |
| Agoraphobia |  |  |  |
| Current (Past 6 Months) | 1 (1.3) | 1 (1.9) | 0 (0.0) |
| Social Anxiety Disorder |  |  |  |
| Current (Past month) | 3 (4.0) | 3 (5.6) | 2 (4.2) |
| Obsessive-Compulsive Disorder |  |  |  |
| Current (Past month) | 1 (1.3) | 0 (0.0) | 0 (0.0) |
| Posttraumatic Stress Disorder |  |  |  |
| Current (Past month) | 3 (4.0) | 2 (3.7) | 0 (0.0) |
| Generalized Anxiety Disorder |  |  |  |
| Current (Past 6 Months) | 6 (8.0) | 2 (3.7) | 0 (0.0) |
| Alcohol Use Disorder |  |  |  |
| Past 12 Months | 1 (1.3) | 1 (1.9) | 0 (0.0) |
| Substance Use Disorder |  |  |  |
| Past 12 Months | 0 (0.0) | 1 (1.9) | 0 (0.0) |

^a^Participants meeting criteria for Recurrent Depressive Disorder may also meet criteria for Current (2 weeks) and Past Major Depressive Disorder.

^b^Participants meeting criteria for Current (2 weeks) Panic Disorder may also meet criteria for Lifetime Panic Disorder.

**Supporting table 13.** Treatment Inventory of Costs in Patients with psychiatric disorders (TIC-P) at baseline and post-treatment

| **Part A: Medical Resource Use** | | | | | | | | | | | | | | | | | | | |
| --- | --- | --- | --- | --- | --- | --- | --- | --- | --- | --- | --- | --- | --- | --- | --- | --- | --- | --- | --- |
|  | | **Baseline, N = 72** | | | | | | | | | **Post-treatment, N = 42** | | | | | | | | |
| Contact with health care provider in the last 4 weeks^a^ (yes/no) | | **Yes (%)** | | | **No (%)** | | **Missing (%)^b^** | | | | **Yes (%)** | | | **No (%)** | | | **Missing (%)^b^** | | |
| Health care provider | |  | | |  | |  | | | |  | | |  | | |  | | |
| General practitioner | | 16 (22.2) | | | 54 (75.0) | | 2 (2.8) | | | | 10 (23.8) | | | 29 (69.0) | | | 3 (7.1) | | |
| Occupational physician | | 1 (1.4) | | | 68 (94.4) | | 3 (4.2) | | | | 0 (0.0) | | | 38 (90.5) | | | 4 (9.5) | | |
| Counsellor | | 4 (5.6) | | | 64 (88.9) | | 4 (5.6) | | | | 2 (4.8) | | | 36 (85.7) | | | 4 (9.5) | | |
| Physiotherapist | | 12 (16.7) | | | 56 (77.8) | | 4 (5.6) | | | | 3 (7.1) | | | 35 (83.3) | | | 4 (9.5) | | |
| Psychiatrist, psychologist or psychotherapist in private practice | | 5 (6.9) | | | 63 (87.5) | | 4 (5.6) | | | | 3 (7.1) | | | 35 (83.3) | | | 4 (9.5) | | |
| Psychiatrist, psychologist or psychotherapist in outpatient department at a psychiatric clinic | | 2 (2.8) | | | 66 (91.7) | | 4 (5.6) | | | | 2 (4.8) | | | 34 (81.0) | | | 6 (14.3) | | |
| Specialist doctor in outpatient department at a general hospital | | 4 (5.6) | | | 64 (88.9) | | 4 (5.6) | | | | 1 (2.4) | | | 36 (85.7) | | | 5 (11.9) | | |
| Specialist doctor in outpatient department at a university hospital | | 4 (5.6) | | | 64 (88.9) | | 4 (5.6) | | | | 1 (2.4) | | | 37 (88.1) | | | 4 (9.5) | | |
| Other health care provider (1) | | 10 (13.9) | | | 56 (77.8) | | 6 (8.3) | | | | 8 (19.0) | | | 30 (71.4) | | | 4 (9.5) | | |
| Other health care provider (2) | | 4 (5.6) | | | 57 (79.2) | | 11 (15.3) | | | | 2 (4.8) | | | 30 (71.4) | | | 10 (23.8) | | |
| If yes to “contact with health care provider in the last 4 weeks”, number of contacts | | **n^c^** | **Mean** | **SD** | | **Min** | | **Max** | | **n^c^** | | **Mean** | | | **SD** | **Min** | | | **Max** |
| General practitioner | | 15 | 1.7 | 1.3 | | 1.0 | | 5.0 | | 10 | | 1.1 | | | 0.3 | 1.0 | | | 2.0 |
| Occupational physician | | 0 | - | - | | - | | - | | 0 | | - | | | - | - | | | - |
| Counsellor | | 4 | 2.0 | 0.8 | | 1.0 | | 3.0 | | 2 | | 1.5 | | | 0.7 | 1.0 | | | 2.0 |
| Physiotherapist | | 12 | 1.7 | 0.9 | | 1.0 | | 4.0 | | 3 | | 2.3 | | | 0.6 | 2.0 | | | 3.0 |
| Psychiatrist, psychologist or psychotherapist in private practice | | 5 | 2.0 | 1.2 | | 1.0 | | 4.0 | | 3 | | 1.3 | | | 0.6 | 1.0 | | | 2.0 |
| Psychiatrist, psychologist or psychotherapist in the outpatient department at a psychiatric clinic | | 2 | 1.0 | 0.0 | | 1.0 | | 1.0 | | 2 | | 1.5 | | | 0.7 | 1.0 | | | 2.0 |
| Specialist doctor in the outpatient department at a general hospital | | 3 | 1.0 | 0.0 | | 1.0 | | 1.0 | | 1 | | 1.0 | | | -^d^ | 1.0 | | | 1.0 |
| Specialist doctor in the outpatient department at a university hospital | | 3 | 1.0 | 0.0 | | 1.0 | | 1.0 | | 1 | | 1.0 | | | -^d^ | 1.0 | | | 1.0 |
| Other health care provider (1) | | 9 | 1.3 | 0.5 | | 1.0 | | 2.0 | | 6 | | 1.3 | | | 0.5 | 1.0 | | | 2.0 |
| Other health care provider (2) | | 3 | 1.3 | 0.6 | | 1.0 | | 2.0 | | 1 | | 1.0 | | | -^d^ | 1.0 | | | 1.0 |
| **Part B: Expenditure and travel in connection with the use of health care^e^** | | | | | | | | | | | | | | | | | | | |
|  |  | **Baseline, N = 31** | | | | | | | | | **Post-treatment, N = 20** | | | | | | | | |
|  |  | **Yes (%)** | | | **No (%)** | | **Missing (%)^b^** | | | | **Yes (%)** | | | **No (%)** | | | **Missing (%)^b^** | | |
| Travel to the health care provider in the last 4 weeks (yes/no) | | 16 (51.6) | | | 14 (45.2) | | 1 (3.2) | | | | 7 (35.0) | | | 13 (65.0) | | | 0 (0.0) | | |
| If yes to “travel to the health care provider in the last 4 weeks”, type of travel^a^ | |  | | |  | |  | | | |  | | |  | | |  | | |
| Car | | 14 (87.5) | | | 2 (12.5) | | 0 (0.0) | | | | 6 (85.7) | | | 1 (14.3) | | | 0 (0.0) | | |
| Taxi | | 0 (0.0) | | | 16 (100.0) | | 0 (0.0) | | | | 1 (14.3) | | | 6 (85.7) | | | 0 (0.0) | | |
| Bus | | 1 (6.3) | | | 15 (93.8) | | 0 (0.0) | | | | 1 (14.3) | | | 6 (85.7) | | | 0 (0.0) | | |
| Train | | 2 (12.5) | | | 14 (87.5) | | 0 (0.0) | | | | 0 (0.0) | | | 7 (100.0) | | | 0 (0.0) | | |
| Other | | 0 (0.0) | | | 16 (100.0) | | 0 (0.0) | | | | 0 (0.0) | | | 7 (100.0) | | | 0 (0.0) | | |
|  | | **n^c^** | **Mean** | **SD** | | **Min** | | **Max** | | **n^c^** | | **Mean** | | | **SD** | **Min** | | | **Max** |
| Total expenses for health care provider not including travelling in SEK(EUR)^f^ | | 27 | 418.5 (41.9) | 500.2 (50.0) | | 0.0 (0.0) | | 2000.0 (200.0) | | 17 | | 308.8 (30.9) | | | 518.8 (51.9) | 0.0 (0.0) | | | 2000.0 (20.00) |
| If yes to “travel to health care provider in the last 4 weeks by car”, total distance driven with car (kilometers) | | 14 | 57.5 | 41.1 | | 5.0 | | 120.0 | | 6 | | 60.8 | | | 75.9 | 5.0 | | | 210.0 |
| If yes to “travel to health care provider in the last 4 weeks”, total expenses for travelling in SEK(EUR)^f^ | | 13 | 215.7 (21.6) | 164.0 (16.4) | | 30.0 (3.0) | | 500.0 (50.0) | | 6 | | 611.7 (61.2) | | | 1184.7 (118.5) | 0.0 (0.0) | | | 3000.0 (300.0) |
| Total time spent in association to healthcare visits (hours) | | 29 | 5.2 | 9.0 | | 0.0 | | 48.0 | | 18 | | 2.1 | | | 2.8 | 0.0 | | | 10.0 |
| Of total time spent in association to healthcare visits, total time spent travelling (hours) | | 27 | 2.2 | 5.7 | | 0.0 | | 30.0 | | 18 | | 0.8 | | | 1.5 | 0.0 | | | 6.0 |
| **Part C: Other help** | | | | | | | | | | | | | | | | | | | |
|  | | **Baseline, N = 72** | | | | | | | | | **Post-treatment, N = 42** | | | | | | | | |
|  | | **Yes (%)** | | | **No (%)** | | **Missing (%)^b^** | | | | **Yes (%)** | | | **No (%)** | | | **Missing (%)^b^** | | |
| Received other help in the last 4 weeks^a^ (yes/no) | |  | | |  | |  | | | |  | | |  | | |  | | |
| Home care | | 0 (0.0) | | | 68 (94.4) | | 4 (5.6) | | | | 0 (0.0) | | | 35 (83.3) | | | 7 (16.7) | | |
| District nurse | | 1 (1.4) | | | 67 (93.1) | | 4 (5.6) | | | | 0 (0.0) | | | 35 (83.3) | | | 7 (16.7) | | |
| Alternative healer practitioner | | 1 (1.4) | | | 66 (91.7) | | 5 (6.9) | | | | 3 (7.1) | | | 32 (76.2) | | | 7 (16.7) | | |
| Self-help group | | 1 (1.4) | | | 66 (91.7) | | 5 (6.9) | | | | 0 (0.0) | | | 35 (83.3) | | | 7 (16.7) | | |
| Help from partner | | 8 (11.1) | | | 59 (81.9) | | 5 (6.9) | | | | 4 (9.5) | | | 31 (73.8) | | | 7 (16.7) | | |
| Help from parents | | 8 (11.1) | | | 59 (81.9) | | 5 (6.9) | | | | 2 (4.8) | | | 33 (78.6) | | | 7 (16.7) | | |
| Help from relatives and friends | | 5 (6.9) | | | 62 (86.1) | | 5 (6.9) | | | | 3 (7.1) | | | 32 (76.2) | | | 7 (16.7) | | |
| Help from others (1) | | 1 (1.4) | | | 63 (87.5) | | 8 (11.1) | | | | 0 (0.0) | | | 34 (81.0) | | | 8 (19.0) | | |
| Help from others (2) | | 0 (0.0) | | | 64 (88.9) | | 8 (11.1) | | | | 0 (0.0) | | | 34 (81.0) | | | 8 (19.0) | | |
| If yes to “received other help in the last 4 weeks”, amount of other help received (hours) | | **n^c^** | **Mean** | **SD** | | **Min** | | **Max** | | **n^c^** | | **Mean** | | | **SD** | **Min** | | | **Max** |
| Home care | | 0 | - | - | | - | | - | | 0 | | - | | | - | - | | | - |
| District nurse | | 1 | 1.0 | -^d^ | | 1.0 | | 1.0 | | 0 | | - | | | - | - | | | - |
| Alternative healer practitioner | | 1 | 1.5 | -^d^ | | 1.5 | | 1.5 | | 2 | | 2.0 | | | 0.0 | 2.0 | | | 2.0 |
| Self-help group | | 1 | 5.0 | -^d^ | | 5.0 | | 5.0 | | 0 | | - | | | - | - | | | - |
| Help from partner | | 4 | 11.0 | 7.7 | | 2.0 | | 20.0 | | 2 | | 18.0 | | | 14.1 | 8.0 | | | 28.0 |
| Help from parents | | 7 | 12.4 | 17.3 | | 1.0 | | 50.0 | | 1 | | 4.0 | | | -^d^ | 4.0 | | | 4.0 |
| Help from relatives and friends | | 5 | 3.8 | 1.5 | | 2.0 | | 6.0 | | 3 | | 2.3 | | | 1.5 | 1.0 | | | 4.0 |
| Help from others (1) | | 1 | 3.0 | -^d^ | | 3.0 | | 3.0 | | 0 | | - | | | - | - | | | - |
| Help from others (2) | | 0 | - | - | | - | | - | | 0 | | - | | | - | - | | | - |
| **Part D: Expenditure and travel in connection with the use of other help^g^** | | | | | | | | | | | | | | | | | | | |
|  | | **Baseline, N = 18** | | | | | | | | | **Post-treatment, N = 8** | | | | | | | | |
|  | | **Yes (%)** | | | **No (%)** | | **Missing (%)^b^** | | | | **Yes (%)** | | | **No (%)** | | | **Missing (%)^b^** | | |
| Travel to other help provider in the last 4 weeks (yes/no) | | 2 (11.1) | | | 12 (66.7) | | 4 (22.2) | | | | 1 (12.5) | | | 7 (87.5) | | | 0 (0.0) | | |
| If yes to “travel to other help provider in the last 4 weeks”, type of travel to other help provider in the last 4 weeks^a^ (yes/no) | |  | | |  | |  | | | |  | | |  | | |  | | |
| Car | | 2 (100.0) | | | 0 (0.0) | | 0 (0.0) | | | | 1 (100.0) | | | 0 (0.0) | | | 0 (0.0) | | |
| Taxi | | 0 (0.0) | | | 2 (100.0) | | 0 (0.0) | | | | 0 (0.0) | | | 1 (100.0) | | | 0 (0.0) | | |
| Bus | | 0 (0.0) | | | 2 (100.0) | | 0 (0.0) | | | | 0 (0.0) | | | 1 (100.0) | | | 0 (0.0) | | |
| Train | | 0 (0.0) | | | 2 (100.0) | | 0 (0.0) | | | | 0 (0.0) | | | 1 (100.0) | | | 0 (0.0) | | |
| Other | | 0 (0.0) | | | 2 (100.0) | | 0 (0.0) | | | | 0 (0.0) | | | 1 (100.0) | | | 0 (0.0) | | |
|  | | **n^c^** | **Mean** | **SD** | | **Min** | | **Max** | | **n^c^** | | **Mean** | | | **SD** | **Min** | | | **Max** |
| Total expenses for other help provider not including travelling in SEK(EUR)^f^ | | 12 | 250.0 (25.0) | 621.6 (62.2) | | 0.0 (0.0) | | 2000 (200.0) | | 5 | | 0.0 (0.0) | | | 0.0 (0.0) | 0.0 (0.0) | | | 0.0 (0.0) |
| If yes to “travel to other help provider in the last 4 weeks by car”, total distance driven with car (kilometers) | | 2 | 19.0 | 15.6 | | 8.0 | | 30.0 | | 1 | | 65.0 | | | -^d^ | 65.0 | | | 65.0 |
| If yes to “travel to other help provider in the last 4 weeks”, total expenses for travelling in SEK(EUR)^f^ | | 2 | 34.0 (3.4) | 15.6 (1.6) | | 23 (2.30) | | 45 (4.50) | | 1 | | 150.0 (15.0) | | | -^d^ | 150.0 (15.0) | | | 150.0 (15.0) |
| If yes to “travel to other help provider in the last 4 weeks”, total time spent travelling (hours) | | 2 | 0.6 | 0.6 | | 0.1 | | 1.0 | | 1 | | 1.0 | | | -^d^ | 1.0 | | | 1.0 |
| **Part E: Use of medication** | | | | | | | | | | | | | | | | | | | |
|  | | **Baseline, N = 72** | | | | | | | | | **Post-treatment, N = 42** | | | | | | | | |
|  |  | **Yes (%)** | | | **No (%)** | | **Missing (%)^b^** | | | | **Yes (%)** | | | **No (%)** | | | **Missing (%)^b^** | | |
| Used medication in the last 4 weeks (yes/no) | | 40 (55.6) | | | 30 (41.7) | | 2 (2.8) | | | | 24 (57.1) | | | 18 (42.9) | | | 0 (0.0) | | |
| If yes to “used medication in the last 4 weeks”, type of medication^h,i^ | |  | | |  | |  | |  | |  | | |  | | |  |  | |
| Antigout agents | | 1 (2.5) | | | 39 (97.5) | | 0 (0.0) | | | | 0 (0.0) | | | 22 (91.7) | | | 2 (8.3) | | |
| Adrenergic bronchodilators | | 1 (2.5) | | | 39 (97.5) | | 0 (0.0) | | | | 0 (0.0) | | | 22 (91.7) | | | 2 (8.3) | | |
| Angiotensin Converting Enzyme Inhibitors | | 1 (2.5) | | | 39 (97.5) | | 0 (0.0) | | | | 0 (0.0) | | | 22 (91.7) | | | 2 (8.3) | | |
| Angiotensin receptor blockers | | 1 (2.5) | | | 39 (97.5) | | 0 (0.0) | | | | 1 (4.2) | | | 21 (87.5) | | | 2 (8.3) | | |
| Antacids | | 2 (5.0) | | | 38 (95.0) | | 0 (0.0) | | | | 1 (4.2) | | | 21 (87.5) | | | 2 (8.3) | | |
| Antidepressant | | 1 (2.5) | | | 39 (97.5) | | 0 (0.0) | | | | 0 (0.0) | | | 22 (91.7) | | | 2 (8.3) | | |
| Antihistamine | | 3 (7.5) | | | 37 (92.5) | | 0 (0.0) | | | | 4 (16.7) | | | 18 (75.0) | | | 2 (8.3) | | |
| Antimigraine agents | | 1 (2.5) | | | 39 (97.5) | | 0 (0.0) | | | | 0 (0.0) | | | 22 (91.7) | | | 2 (8.3) | | |
| Asthma medication | | 1 (2.5) | | | 39 (97.5) | | 0 (0.0) | | | | 1 (4.2) | | | 21 (87.5) | | | 2 (8.3) | | |
| Betahistine | | 0 (0.0) | | | 40 (100.0) | | 0 (0.0) | | | | 1 (4.2) | | | 21 (87.5) | | | 2 (8.3) | | |
| Birth control | | 2 (5.0) | | | 38 (95.0) | | 0 (0.0) | | | | 0 (0.0) | | | 22 (91.7) | | | 2 (8.3) | | |
| Blood pressure medication | | 5 (12.5) | | | 35 (87.5) | | 0 (0.0) | | | | 2 (8.3) | | | 20 (83.3) | | | 2 (8.3) | | |
| Bronchodilator combinations | | 1 (2.5) | | | 39 (97.5) | | 0 (0.0) | | | | 0 (0.0) | | | 22 (91.7) | | | 2 (8.3) | | |
| B-vitamin medication | | 2 (5.0) | | | 38 (95.0) | | 0 (0.0) | | | | 0 (0.0) | | | 22 (91.7) | | | 2 (8.3) | | |
| Calcium channel blocking agents | | 1 (2.5) | | | 39 (97.5) | | 0 (0.0) | | | | 1 (4.2) | | | 21 (87.5) | | | 2 (8.3) | | |
| Dandruff medication | | 1 (2.5) | | | 39 (97.5) | | 0 (0.0) | | | | 0 (0.0) | | | 22 (91.7) | | | 2 (8.3) | | |
| Diabetes medicine | | 1 (2.5) | | | 39 (97.5) | | 0 (0.0) | | | | 1 (4.2) | | | 21 (87.5) | | | 2 (8.3) | | |
| Dipeptidyl peptidase 4 inhibitors | | 0 (0.0) | | | 40 (100.0) | | 0 (0.0) | | | | 1 (4.2) | | | 21 (87.5) | | | 2 (8.3) | | |
| Fatty acid derivative anticonvulsants | | 1 (2.5) | | | 39 (97.5) | | 0 (0.0) | | | | 0 (0.0) | | | 22 (91.7) | | | 2 (8.3) | | |
| Glucocorticoids | | 1 (2.5) | | | 39 (97.5) | | 0 (0.0) | | | | 1 (4.2) | | | 21 (87.5) | | | 2 (8.3) | | |
| Immunosuppression | | 1 (2.5) | | | 39 (97.5) | | 0 (0.0) | | | | 0 (0.0) | | | 22 (91.7) | | | 2 (8.3) | | |
| Mast cell stabilizers | | 1 (2.5) | | | 39 (97.5) | | 0 (0.0) | | | | 0 (0.0) | | | 22 (91.7) | | | 2 (8.3) | | |
| Miscellaneous analgesics | | 9 (22.5) | | | 31 (77.5) | | 0 (0.0) | | | | 5 (20.8) | | | 17 (70.8) | | | 2 (8.3) | | |
| Miscellaneous anxiolytics, sedatives and hypnotics | | 1 (2.5) | | | 39 (97.5) | | 0 (0.0) | | | | 2 (8.3) | | | 20 (83.3) | | | 2 (8.3) | | |
| Nasal steroids | | 0 (0.0) | | | 40 (100.0) | | 0 (0.0) | | | | 1 (4.2) | | | 21 (87.5) | | | 2 (8.3) | | |
| Nonsteroidal anti-inflammatory drugs | | 18 (45.0) | | | 22 (55.0) | | 0 (0.0) | | | | 7 (29.2) | | | 15 (62.5) | | | 2 (8.3) | | |
| Non-sulfonylureas | | 1 (2.5) | | | 39 (97.5) | | 0 (0.0) | | | | 1 (4.2) | | | 21 (87.5) | | | 2 (8.3) | | |
| Ophthalmic steroids with anti-infectives | | 0 (0.0) | | | 40 (100.0) | | 0 (0.0) | | | | 1 (4.2) | | | 21 (87.5) | | | 2 (8.3) | | |
| Penicillin | | 1 (2.5) | | | 39 (97.5) | | 0 (0.0) | | | | 0 (0.0) | | | 22 (91.7) | | | 2 (8.3) | | |
| Proton pump inhibitors | | 2 (5.0) | | | 38 (95.0) | | 0 (0.0) | | | | 1 (4.2) | | | 21 (87.5) | | | 2 (8.3) | | |
| Salicylates | | 1 (2.5) | | | 39 (97.5) | | 0 (0.0) | | | | 0 (0.0) | | | 22 (91.7) | | | 2 (8.3) | | |
| Selective immunosuppressants | | 1 (2.5) | | | 39 (97.5) | | 0 (0.0) | | | | 1 (4.2) | | | 21 (87.5) | | | 2 (8.3) | | |
| Selective serotonin reuptake inhibitors | | 7 (17.5) | | | 33 (82.5) | | 0 (0.0) | | | | 3 (12.5) | | | 19 (79.2) | | | 2 (8.3) | | |
| Skeletal muscle relaxants | | 2 (5.0) | | | 38 (95.0) | | 0 (0.0) | | | | 1 (4.2) | | | 21 (87.5) | | | 2 (8.3) | | |
| St. John's wort | | 1 (2.5) | | | 39 (97.5) | | 0 (0.0) | | | | 0 (0.0) | | | 22 (91.7) | | | 2 (8.3) | | |
| Statins | | 1 (2.5) | | | 39 (97.5) | | 0 (0.0) | | | | 1 (4.2) | | | 21 (87.5) | | | 2 (8.3) | | |
| Thyroid drugs | | 3 (7.5) | | | 37 (92.5) | | 0 (0.0) | | | | 1 (4.2) | | | 21 (87.5) | | | 2 (8.3) | | |
| Topical steroids | | 1 (2.5) | | | 39 (97.5) | | 0 (0.0) | | | | 0 (0.0) | | | 22 (91.7) | | | 2 (8.3) | | |
| Vitamin supplement | | 1 (2.5) | | | 39 (97.5) | | 0 (0.0) | | | | 0 (0.0) | | | 22 (91.7) | | | 2 (8.3) | | |
|  | | **n^c^** | **Mean** | **SD** | | **Min** | | **Max** | | **n^c^** | | **Mean** | | | **SD** | **Min** | | | **Max** |
| Time used medication in the last 4 weeks (days)^j^ | | - | - | - | | - | | - | | - | | - | | | - | - | | | - |
| If yes to “used medication in the last 4 weeks”, total expenses for medication in SEK(EUR)^f^ | | 34 | 85.4 (8.5) | 110.0 (11.9) | | 0.0 (0.0) | | 450.0 (45.0) | | 19 | | 235.3 (23.5) | | | 581.8 (58.2) | 0.0 (0.0) | | | 2600 (260.0) |
| **Part F: Employment/work** | | | | | | | | | | | | | | | | | | | |
|  | | **Baseline, N = 72** | | | | | | | | | **Post-treatment, N = 42** | | | | | | | | |
|  | | **Yes (%)** | | | **No (%)** | | **Missing (%)^b^** | | | | **Yes (%)** | | | **No (%)** | | | **Missing (%)^b^** | | |
| Currently employed (yes/no) | | 60 (83.3) | | | 11 (15.3) | | 1 (1.4) | | | | 37 (88.1) | | | 5 (11.9) | | | 0 (0.0) | | |
|  | | **n^c^** | **Mean** | **SD** | | **Min** | | **Max** | | **n^c^** | | **Mean** | | | **SD** | **Min** | | | **Max** |
| If yes to “currently employed”, time working per week (hours) | | 59 | 38.3 | 7.2 | | 10.0 | | 60.0 | | 36 | | 40.2 | | | 5.8 | 26.0 | | | 60.0 |
| If yes to “currently employed”, weekly income in SEK(EUR)^f^ | | 57 | 11604.0 (1160.4) | 9167.7 (916.8) | | 2500.0 (250.0) | | 50000.0 (5000.0) | | 32 | | 11242.7 (1124.3) | | | 6971.5 (697.2) | 5000.0  (500.0) | | | 45000.0 (4500.0) |
| **Part G: Impaired working capacity and sick leave** | | | | | | | | | | | | | | | | | | | |
|  | | **Baseline, N = 72** | | | | | | | | | **Post-treatment, N = 42** | | | | | | | | |
|  | | **Yes (%)** | | | **No (%)** | | **Missing (%)^b^** | | | | **Yes (%)** | | | **No (%)** | | | **Missing (%)^b^** | | |
| Sick leave for 5 or more consecutive months in the last 12 months^a^ (yes/no) | | 11 (15.3) | | | 61 (84.7) | | 0 (0.0) | | | | 6 (14.3) | | | 35 (83.3) | | | 1 (2.4) | | |
| If yes, full-time | | 8 (72.7) | | | 3 (27.3) | | 0 (0.0) | | | | 5 (83.3) | | | 1 (16.7) | | | 0 (0.0) | | |
| If yes, part-time | | 9 (81.8) | | | 2 (18.2) | | 0 (0.0) | | | | 4 (66.7) | | | 2 (33.3) | | | 0 (0.0) | | |
| Sick leave in the last 4 weeks (yes/no) | | 11 (15.3) | | | 61 (84.7) | | 0 (0.0) | | | | 6 (14.3) | | | 36 (85.7) | | | 0 (0.0) | | |
| Sick leave currently (yes/no) | | 6 (54.5) | | | 5 (45.5) | | 0 (0.0) | | | | 5 (83.3) | | | 1 (16.7) | | | 0 (0.0) | | |
| If yes, full-time sick leave | | 4 (66.7) | | | 2 (33.3) | | 0 (0.0) | | | | 2 (40.0) | | | 3 (60.0) | | | 0 (0.0) | | |
| If yes, part-time sick leave | | 2 (33.3) | | | 4 (66.7) | | 0 (0.0) | | | | 3 (60.0) | | | 2 (40.0) | | | (0 (0.0) | | |
| Been working while not feeling well in the last 4 weeks (yes/no)^k^ | | 6 (8.3) | | | 6 (8.3) | | 60 (83.3) | | | | 0 (0.0) | | | 7 (17.7) | | | 42 (83.3) | | |
|  | | **n^c^** | **Mean** | **SD** | | **Min** | | **Max** | | **n^c^** | | **Mean** | | | **SD** | **Min** | | | **Max** |
| If yes to “full-time sick leave for 5 or more consecutive months in the last 12 months”, length of full-time sick leave (month) | | 8 | 5.4 | 2.4 | | 2.0 | | 10.0 | | 5 | | 4.8 | | | 2.7 | 1.0 | | | 7.0 |
| If yes to “part-time sick leave for 5 or more consecutive months in the last 12 months”, length of part-time sick leave (month) | | 8 | 4.5 | 3.9 | | 1.0 | | 12.0 | | 3 | | 2.7 | | | 2.9 | 1.0 | | | 6.0 |
| If yes, part-time sick leave percentage (%) | | 4 | 50.0 | 20.4 | | 25.0 | | 75.0 | | 2 | | 62.5 | | | 17.7 | 50.0 | | | 75.0 |
| If yes to “sick leave in the last 4 weeks”, length of time sick leave (days) | | 9 | 7.8 | 6.3 | | 2.0 | | 20.0 | | 4 | | 15.8 | | | 11.8 | 3.0 | | | 30.0 |
| If yes to “currently part-time sick leave”, part-time sick leave percentage (%) | | 2 | 42.5 | 24.7 | | 25 | | 60.0 | | 3 | | 41.7 | | | 14.4 | 25.0 | | | 50.0 |
| If yes to “been working while not feeling well in the last 4 weeks”, length of time working when not feeling well (days) | | 5 | 6.0 | 5.2 | | 2.0 | | 15.0 | | 0 | | - | | | - | - | | | - |
| Amount of work done on days working not feeling well^k,l,m^ | | 2 | 7.5 | 3.5 | | 5.0 | | 10.0 | | 2 | | 8.0 | | | 2.8 | 6.0 | | | 10.0 |
| **Part H: Housework and leisure time^m^** | | | | | | | | | | | | | | | | | | | |
|  | | **Baseline, N = 72** | | | | | | | | | **Post-treatment, N = 42** | | | | | | | | |
|  | | **Yes (%)** | | | **No (%)** | | **Missing (%)** | | | | **Yes (%)** | | **No (%)** | | | | **Missing (%)** | | |
| Had days not able to do anything in the household in the last 4 weeks (yes/no) | | 1 (1.4) | | | 2 (2.8) | | 69 (95.8) | | | | 1 (2.4) | | 1 (2.4) | | | | 40 (95.2) | | |
| Had days carried out housework when not feeling well in the last 4 weeks (yes/no) | | 2 (2.8) | | | 1 (1.4) | | 69 (95.8) | | | | 1 (2.4) | | 1 (2.4) | | | | 40 (95.2) | | |
| Had days when not been able to pursue normal leisure activities in the last 4 weeks (yes/no) | | 2 (2.8) | | | 1 (1.4) | | 69 (95.8) | | | | 1 (2.4) | | 1 (2.4) | | | | 40 (95.2) | | |
| Had days when pursued normal leisure activities when not feeling well in the last 4 weeks (yes/no) | | 1 (1.4) | | | 2 (2.8) | | 69 (95.8) | | | | 2 (4.8) | | 0 (0.0) | | | | 40 (95.2) | | |
|  | | **n^c^** | **Mean** | **SD** | | **Min** | | **Max** | | **n^c^** | | **Mean** | | | **SD** | **Min** | | | **Max** |
| Length of time per week usually spent on housework (hours) | | 3 | 25.0 | 20.0 | | 5.0 | | 45.0 | | 2 | | 4.3 | | | 3.9 | 1.5 | | | 7.0 |
| If yes to “had days not able to do anything in the household in the last 4 weeks”, length of time not able to do any housework (days) | | 1 | 5.0 | -^d^ | | 5.0 | | 5.0 | | 1 | | 4.0 | | | -^d^ | 4.0 | | | 4.0 |
| If yes to “had days carried out housework when not feeling well in the last 4 weeks”, length of time done housework when not feeling well (days) | | 2 | 2.5 | 0.7 | | 2.0 | | 3.0 | | 1 | | 5.0 | | | -^d^ | 5.0 | | | 5.0 |
| Amount of housework done during a day when not feeling well, compared to a day when feeling well (Likert scale 0-10)^m^ | | 2 | 3.5 | 2.1 | | 2.0 | | 5.0 | | 2 | | 10.0 | | | 0.0 | 10.0 | | | 10.0 |
| If yes to “had days when not able to pursue normal leisure activities in the last 4 weeks”, length of times not being able to pursue normal leisure activities (days) | | 2 | 7.5 | 6.4 | | 3.0 | | 12.0 | | 1 | | 3.0 | | | -^d^ | 3.0 | | | 3.0 |
| If yes to “had days when pursued normal leisure activities when not feeling well in the last 4 weeks”, length of time pursued normal leisure activities when not feeling well (days) | | 1 | 2.0 | -^d^ | | 2.0 | | 2.0 | | 2 | | 15.5 | | | 17.7 | 3.0 | | | 28.0 |
| If yes to “had days when pursued normal leisure activities when not feeling well in the last 4 weeks”, amount of time devoted to normal leisure activities during a day when not feeling well, compared to a day when feeling well (Likert scale 0-10)^o^ | | 1 | 0.0 | -^d^ | | 0.0 | | 0.0 | | 2 | | 8.5 | | | 2.1 | 7.0 | | | 10.0 |

Abbreviations: EUR, Euros, SE, Swedish Kronas, TIC-P, Treatment Inventory of Costs in Patients with psychiatric disorders (TIC-P).

Percentages may not always total 100 due to rounding.

^a^ Multiple responses possible.

^b^Missing may indicate missing self-report data or missing data due to researcher error.

^c^Numbers may be smaller than expected due to missing data.

^d^Standard deviation cannot be calculated, sample only contains one value.

^e^Part B is only relevant to those participants who responded yes to part A.

^f^1 SEK = 0.10 €.

^g^Part D only relevant to those participants who responded yes to part C.

^h^Participants can multiple several types of medication during 4 weeks.

^i^Type of medication is self-report data and medication types have been categorised where possible in accordance with classification systems from <https://www.drugs.com/> .

^j^Time used medication in the last 4 weeks (days) is not reported due to inconsistent answers.

^k^Question was not presented correctly on the online assessments via the portal due to researcher errors. Data is presented for participants who completed the TIC-P over the telephone at baseline and post-treatment with a member of the research team and for participants answering yes to “Sick leave in the last 4 weeks”.

^l^Likert scale: 0 = "Done nothing"; 10 = "Done as much as a day when I felt well”.

^m^Part H was not presented in online assessments via the Portal due to researcher error. Data is presented only for participants who completed the TIC-P over the telephone at baseline and post-treatment (12 weeks) with a member of the research team.

^n^Likert scale: 0 = “Did not do anything at all"; 10 = completed just as much compared to a day when I felt well”.

^o^Likert scale: 0 = “Could not devote any time to pursuing my normal hobbies”; 10 = “Devoted just as much time as much compared to a day when I felt well”.
