# Supplementary figures and images for: Internet‐administered, low‐intensity cognitive behavioral therapy for parents of children treated for cancer: A feasibility trial (ENGAGE)
Source: Cancer Med. 2022 Nov 20;12(5):6225–43. doi: 10.1002/cam4.5377 (PMC10028033; doi:10.1002/cam4.5377)

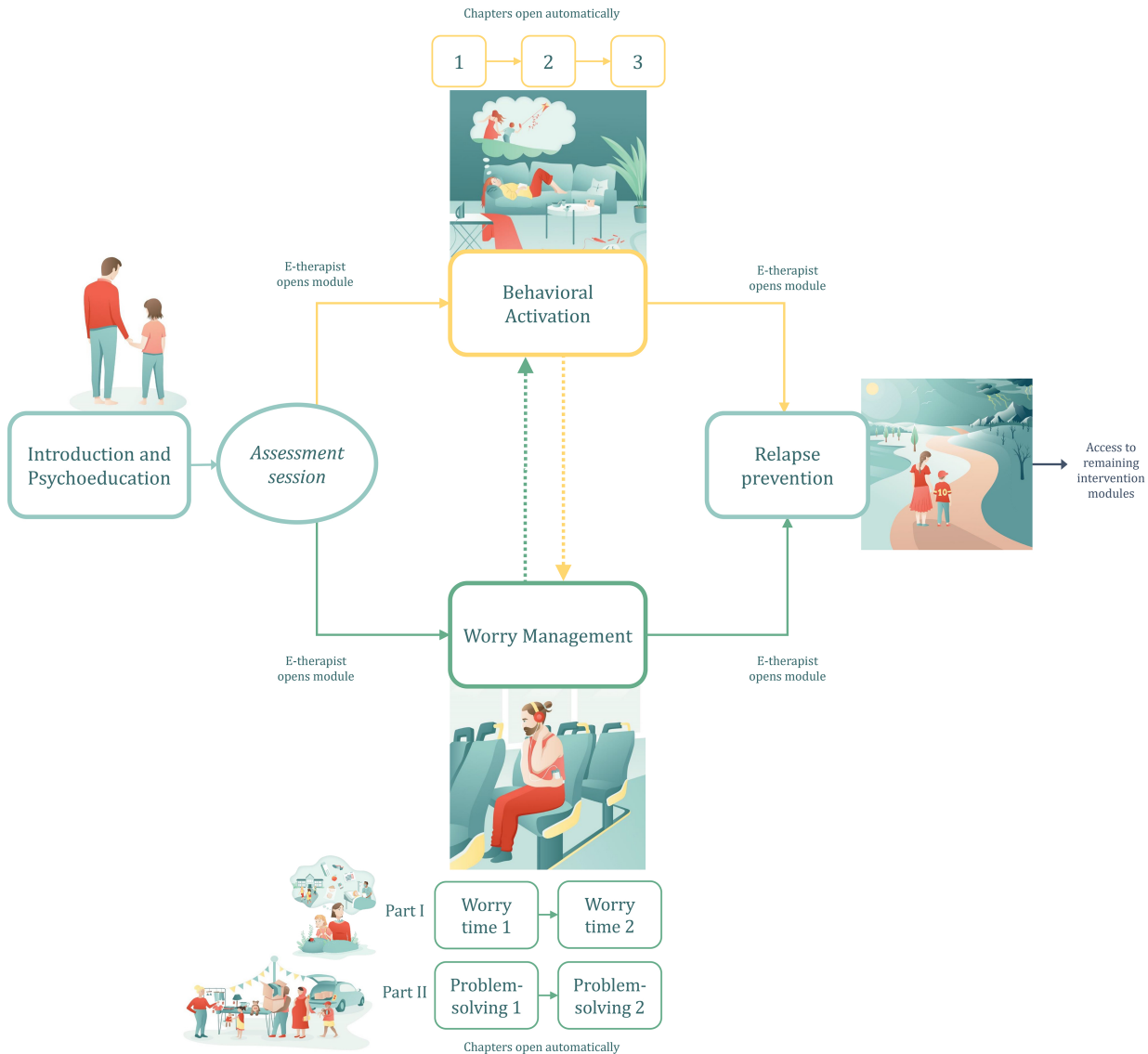

Supplement: Supplementary file 1 — Data S1 [file CAM4-12-6225-s001.zip › CAM4_5377_Supporting_figure_1.pdf]
